# Metal Oxide Based Heterojunctions for Gas Sensors: A Review

**DOI:** 10.3390/nano11041026

**Published:** 2021-04-17

**Authors:** Shulin Yang, Gui Lei, Huoxi Xu, Zhigao Lan, Zhao Wang, Haoshuang Gu

**Affiliations:** 1 Hubei Key Laboratory for Processing and Application of Catalytic Materials, School of Physics and Electronic Information, Huanggang Normal University, Huanggang 438000, China; yangsl@hgnu.edu.cn (S.Y.); leig@hgnu.edu.cn (G.L.); lanzhg@hgnu.edu.cn (Z.L.); 2 Hubei Key Laboratory of Ferro & Piezoelectric Materials and Devices, Faculty of Physics and Electronic Sciences, Hubei University, Wuhan 430062, China; wangzhao@hubu.edu.cn

**Keywords:** metal oxide, heterojunctions, gas sensor, sensing mechanism, review

## Abstract

The construction of heterojunctions has been widely applied to improve the gas sensing performance of composites composed of nanostructured metal oxides. This review summarises the recent progress on assembly methods and gas sensing behaviours of sensors based on nanostructured metal oxide heterojunctions. Various methods, including the hydrothermal method, electrospinning and chemical vapour deposition, have been successfully employed to establish metal oxide heterojunctions in the sensing materials. The sensors composed with the built nanostructured heterojunctions were found to show enhanced gas sensing performance with higher sensor responses and shorter response times to the targeted reducing or oxidising gases compare with those of the pure metal oxides. Moreover, the enhanced gas sensing mechanisms of the metal oxide-based heterojunctions to the reducing or oxidising gases are also discussed, with the main emphasis on the important role of the potential barrier on the accumulation layer.

## 1. Introduction

Gas sensors based on nanostructured metal oxides have attracted significant interest over the last few decades due to their advantages of low cost, ease of fabrication, high sensor response and short response/recovery times [1,2,3,4]. Various metal oxides have been successfully assembled as gas sensors since Seiyama et al. reported their research on the gas sensing performance of the ZnO thin film in the 1960s [5,6]. According to the sensing behaviours of the metal oxides, sensing metal oxides are typically divided into two main groups: n-type metal oxides and p-type metal oxides. Normally, the resistances of the n-type metal oxides decrease (or increase) towards reducing gases such as H_2_, H_2_S, CO, CH_4_, NH_3_ and other volatile organic compounds (or oxidising gases such as NO_2_, NO, O_3_, SO_2_, etc.), while p-type metal oxides exhibit the opposite behaviour [7,8]. The n-type metal oxides of SnO_2_, TiO_2_, WO_3_, MoO_3_, Nb_2_O_5_, ZnO, etc., and the p-type metal oxides of CuO, Co_3_O_4_, Cr_2_O_3_, NiO, PdO, etc., have been widely studied for their gas sensing behaviours towards both reducing and oxidizing gases [9,10,11,12].

**Figure 1:**
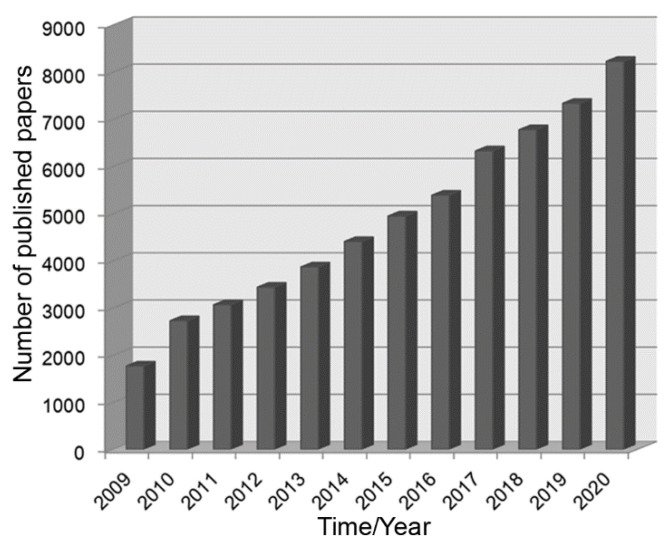
The number of the published papers on nanostructured metal oxide heterojunctions for high-performance gas sensors during 2009–2020 as obtained from the Web of Science. The words “nanostructured metal oxide heterojunctions” or “high-performance gas sensors” were keyed into the “topic” search box.

In recent years, the advancement of new technologies and methods has induced a boom in nanomaterials. Nanostructured metal oxides with various morphologies, such as nanoparticles, nanosheets, nanowires, nanorods, nanoribbons, nanofibres, nanoflowers and nanocages, have been successfully prepared through the routes of hydrothermal processing, thermal oxidation, sol-gel processing, atomic layer deposition, etc. Nanoscale metal oxides have been reported to exhibit promising gas sensing performances, benefiting from their high specific surface areas [13] and active surface states [14]. Specifically, the sensor response of the pure ZnO nanowires was ~15 towards 0.5 ppm NO_2_ at a working temperature of 225 °C [15]. Other sensors based on SnO_2_ nanowires [16], TiO_2_ nanotubes [17], WO_3_ nanoparticles [18] and In_2_O_3_ nanofibres [19] have also been found to respond to the gases of NO_2_, formaldehyde, H_2_S and CO, respectively. The gas sensing performances of the gas sensors mentioned above could be further improved to better meet the demands of practical applications via compositing the metal oxide with another (different) metal oxide to form a heterojunction between them. It is noteworthy that when the main phase of a metal oxide was decorated or composited with the second phase of a different semiconductor, the interface between them was known as the structure of a heterojunction in the sensing material [20]. Reports show the specific surface area of the composite is higher than that of the pure metal oxide [21], and the modulation of the potential barrier or accumulation layer in the composite effectively improves the gas sensing behaviour [22,23]. More and more researchers have focused their attention on the studies of the high-performance sensors based on nanostructured metal oxide heterojunctions, as shown in Figure 1. For example, the ordered mesoporous WO_3_/ZnO nanocomposites synthesised with a hydrothermal method displayed an enhanced sensor response of 168.7 to 1 ppm NO_2_ at a working temperature of 150 °C, over 10 times higher than that of the pure WO_3_ [24]. Moreover, the sensor response of CeO_2_ nanostructures modified with NiO was reported to be ~1570, much higher than that of the pure CeO_2_ (139). The response time and the recovery time of the composite was 15 s and 19 s, respectively, which is also shorter than that of the pure CeO_2_ (96 s/118 s) [25]. Therefore, the construction of heterojunctions could be a successful method to improve the gas sensing performances of sensors based on metal oxides.

As reported, there have been different kinds of heterojunctions assembled to improve the gas sensing performances of metal oxides, such as n-n, n-p, p-n or p-p heterojunctions [26,27,28,29]. Note that, in this paper, the type of heterojunction is defined according to the dominant material or the main phase in the composite [30,31]. Accordingly, an n-p heterojunction is formed when the main phase of an n-type metal oxide is modified with a second phase of a p-type semiconductor. Similarly, a p-n heterojunction is established through compositing a p-type metal oxide with an n-type semiconductor. An n-n (or p-p) heterojunction would also be constructed if an n-type (p-type) metal oxide is decorated with a different n-type (p-type) semiconductor in the composite. For example, CuO-decorated ZnO or ZnO-decorated WO_3_ are the typical n-p or n-n heterojunctions. The ZnO-decorated CuO or NiO-decorated CuO are defined as the p-n or p-p heterojunctions. With the development in the techniques to synthesise nanomaterials, it is facile to construct heterojunctions in composites composed of metal oxides. The metal oxide-based heterojunctions have been successfully established through various combined technologies, such as the thermal oxidation [28], hydrothermal method [32], electrospinning [33,34], chemical vapour deposition (CVD) [35], pulsed laser deposition (PLD) [36], the co-precipitation method [37] and the solvothermal method [38]. For example, the hydrothermal method was reported to effectively prepare the ZnO/SnO_2_ [39] and the NiO/SnO_2_ composites [32]. The difference in the Fermi levels of the two metal oxides in the obtained composite would lead to the formation of the potential barrier at their interfaces, an important factor for improving sensing property of the gas sensor based on heterojunctions. The hydrothermal method combined with CVD was also used by Li et al. to establish heterojunctions composed of vertically aligned MoS_2_/ZnO nanowires [40]. SnO_2_-CuO heterojunctions were successfully constructed via electrospinning [41]. Their results clearly indicated that the sensor response of the sensor based on the MoS_2_/ZnO nanowires or the SnO_2_-CuO heterojunctions was highly improved compared with that of the bare ZnO or SnO_2_. Though there have been a number of references reviewing the developments in the gas sensing performances of given metal oxides, only a few articles provide a comprehensive review of the effects of the heterojunctions on the enhanced gas sensing performances of composites based on nanostructured metal oxides or separately discuss heterojunctions with n-n, n-p, p-n or p-p structures. Moreover, the enhanced gas sensing mechanism of a given type of heterojunction to a reducing or an oxidising gas should also be studied and summarised. The synthesised methods and the gas sensing performances of the normally studied metal oxides as well as the important roles of the heterojunctions need to be systematically summarised and compared. This will allow us to fully understand the improved gas sensing properties of metal oxide heterojunctions.

In this review, the typical synthetic routes of n-n, n-p, p-n and p-p heterojunctions based on metal oxides are introduced. The gas sensing behaviours of the n-n/n-p heterojunctions (or p-n/p-p heterojunctions) are based on SnO_2_ and TiO_2_. ZnO, WO_3_, MoO_3_, In_2_O_3_, CuO, Cr_2_O_3_, NiO and Co_3_O_4_, etc., semiconductors are reviewed and compared to show the effects of the heterojunctions on the gas sensing performances of the metal oxides. The enhanced gas sensing mechanisms of the composites towards reducing and oxidising gases are also discussed in detail to systematically understand the role of the built heterojunctions in improving the gas sensing properties of the composites.

## 2. Nanostructured Metal Oxide Heterojunctions for High-Performance Gas Sensors

**Figure 2:**
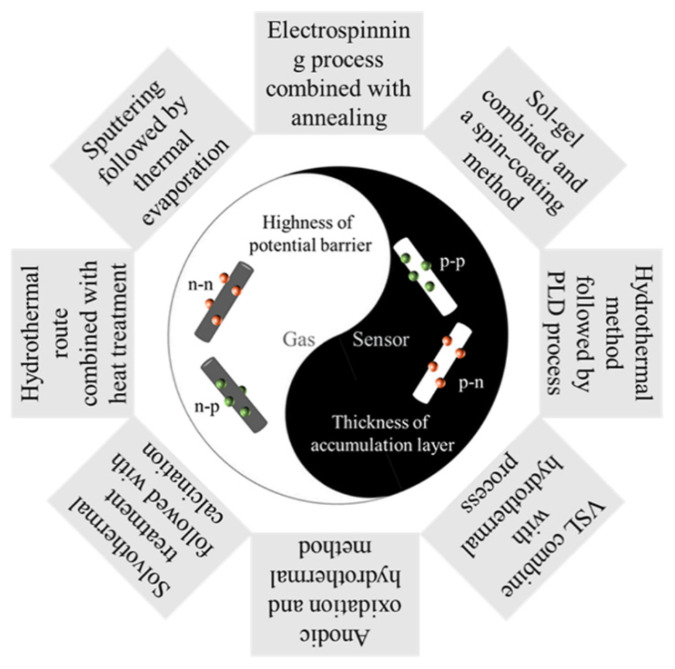
Typical types of heterojunctions for high-performance gas sensors and assembly strategies.

As reported, the formation of heterojunctions could be a positive effective strategy to improve the gas sensing performance of the metal oxides. Various methods such as hydrothermal [42], PLD [36], vapour-liquid-solid (VSL) [43], anodic oxidation [44], solvothermal treatment [45], sputtering [46], thermal evaporation [47], electrospinning [33], sol-gel [48] and spin-coating [49] have been successfully applied to assemble the heterostructures in the sensors, and are generally combined to form various heterojunctions (n-n, n-p, p-n or p-p types), as displayed in Figure 2. Other methods to assemble nanostructured metal oxide heterojunctions are listed in Tables S1–S4 (see Supporting Materials), along with the improved gas sensing performances of sensors based on n-p, n-n, p-n and p-p heterojunctions. Some of the typical nanostructured heterojunctions with the n-type (or p-type) metal oxides as the main phases are discussed in the following sections.

### 2.1. Enhanced Gas Sensing Performances of n-n Junctions or n-p Junctions

Heterojunctions with the n-p or the n-n structure in the sensing materials have been reported to be successful strategies to enhance their gas sensing properties. When the p-type metal oxide (acting as the second phase) is attached to an n-type metal oxide (acting as the main phase), an n-p heterojunction is formed between the two sensing metal oxides. Additionally, n-n heterojunctions can also be assembled in a similar way. One of the common routes to establish the n-n (or n-p) heterojunctions is to prepare the main n-type metal oxides and then decorate the prepared n-type metal oxides with the n-type (or p-type) metal oxides [50]. The modulation of the built potential barrier in the n-n or n-p heterojunction can effectively modify the resistance of the sensing material, and thus greatly improve the gas sensing properties of the sensor composed with the n-n (or n-p) heterojunctions. Meanwhile, it is also noticed that the majority of heterojunctions are assembled as a decorated structure, core-shell structure or mixed structure (one metal oxide mixed with another metal oxide), discussed in the following subsections.

#### 2.1.1. Gas Sensors Based on n-n Junctions

**Figure 3:**
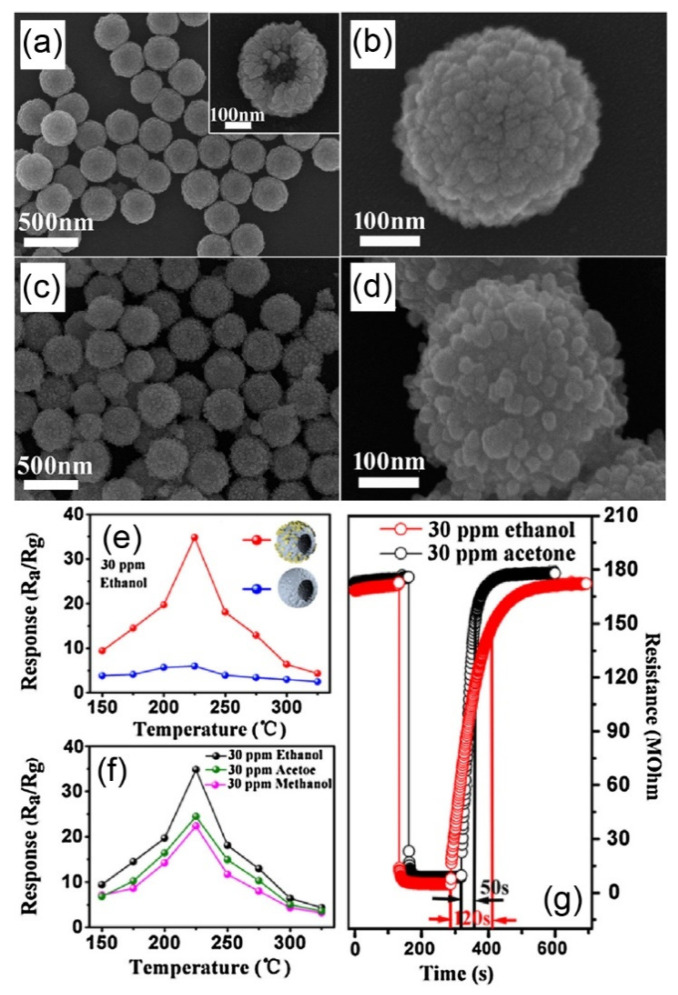
SEM images of the pure SnO_2_ hollow spheres (**a**,**b**) and ZnO-decorated SnO_2_ (n-n junctions) (**c**,**d**), sensor response of pure SnO_2_ hollow spheres and the ZnO-decorated SnO_2_ hollow spheres to 30 ppm ethanol at different operating temperatures (**e**), the sensor response of ZnO-decorated SnO_2_ hollow spheres to 30 ppm ethanol, acetone and methanol at different operating temperatures (**f**), and the dynamic sensing performance of the decorated SnO_2_ hollow spheres towards 30 ppm ethanol or acetone at 225 °C (**g**). Copied with permission from reference [39]. Copyright 2017, Elsevier.

Many references report the improved gas sensing performances of sensors based on n-n heterojunctions. For example, Lu et al. reported the gas sensing properties of the ZnO-decorated SnO_2_ hollow spheres towards the ethanol synthesised via a two-step hydrothermal method [39]. Hollow spheres of SnO_2_ of ~100 nm thickness were synthesised via a facile template-free hydrothermal route (see Figure 3a,b) with ZnO nanoparticles of 10–30 nm diameter (see Figure 3c,d) uniformly decorated on its surface via a solution route. The sensor response of the ZnO-decorated SnO_2_ hollow spheres was calculated to be 34.8 towards 30 ppm ethanol at their optimised operating temperature of 225 °C (see Figure 3e), much higher than that of the bare SnO_2_ (~5.7 times). Their further research indicated that the composite also exhibited promising selectivity to acetone compared with methanol (Figure 3f). The recovery time of the composite towards 30 ppm ethanol (50 s) was also much shorter than that of acetone (120 s) at the same concentrations as shown in Figure 3g. In addition, the SnO_2_ compositing with Co_3_O_4_ and SiO_2_ have been reported to be promising gas sensing materials. The SnO_2_/SiO_2_ heterojunctions were synthesised via a facile method of a magnetron sputtering process and exhibited promising H_2_ sensing performance at room temperature [51]. Hybrid Co_3_O_4_/SnO_2_ core-shell nanospheres prepared with a one-step hydrothermal method demonstrated a measured response of 13.6 to 100 ppm NH_3_ at 200 °C, a value two times higher than that of the solid nanospheres [52]. CeO_2_-decorated ZnO nanosheets were prepared by a hydrothermal process in combination with the wet impregnation method, exhibited an enhanced sensor response of 90 to 100 ppm ethanol at 310 °C [53]. Additionally, Kim et al. have fabricated ZnO-SnO_2_ nanofibres through an electrospinning process to effectively detect CO [54].

The *α*-MoO_3_/TiO_2_ core/shell nanorods have been synthesised through a hydrothermal process combined with the following annealing process in air atmosphere [55]. Uniform *α*-MoO_3_ nanorods were first prepared and then coated with a shell of TiO_2_ via a modified wet-chemical method. It was found that the core/shell nanorods exhibited an improved gas sensing performance to 10 ppm ethanol at 180 °C with a short response time of less than 40 s. Meanwhile, the SnO_2_-core/ZnO-shell nanowires [56] and the Ga_2_O_3_-core/ZnO-shell nanorods [57] were successfully synthesised through a plasma-enhanced CVD and atomic layer deposition (ALD), respectively, which also exhibited promising gas sensing performances. The MoO_3_ nanorods decorated with the ZnO nanoparticles were also reported to be a promising material to detect 100 ppm ethanol with a sensor response of ~30 at the working temperature of 250 °C [58]. Besides the nanocomposites discussed above, it was reported that *α*-MoO_3_ compositing with WO_3_ through a sol-gel method [59] or with Fe_2_O_3_ nanoparticles via a hydrothermal method [60] also showed improved gas sensing performances towards O_2_ or xylene, respectively.

In addition, ZnO nanorods/TiO_2_ nanoparticles [61] and the ZnO/La_0.8_Sr_0.2_Co_0.5_Ni_0.5_O_3_ heterojunction structure [62] were successfully constructed to research their improved gas sensing performances to NO_2_ and CO, respectively. WO_3_ compositing with SnO_2_ was reported to be a potential material to detect acetone [63], while WO_3_-modified ZnO nanoplates synthesised via the hydrothermal route were assembled for the detection of NH_3_ [26]. Other effective methods to synthesise n-n heterojunctions and their gas sensing performances are listed in Table S1.

#### 2.1.2. Gas Sensors Based on n-p Junctions

**Figure 4:**
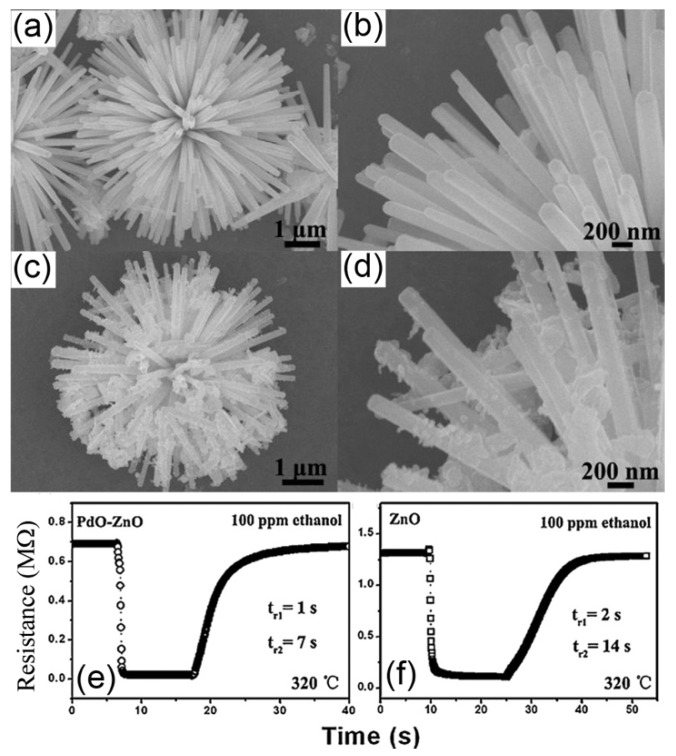
SEM images of pure flower-like ZnO (**a**,**b**) and the PdO nanoparticle-decorated ZnO (n-p junctions) (**c**,**d**), the dynamic sensing performance of the sensor based on the PdO nanoparticle-decorated ZnO (**e**) or pure ZnO (**f**) to 100 ppm ethanol at 320 °C. Copied with permission [42]. Copyright 2013, Elsevier.

The sensors based on the n-p heterojunctions have been found to show promising gas sensing properties towards various gases. For example, PdO nanoparticles-decorated flower-like ZnO structures (see Figure 4) were prepared by Zhang et al. through a surfactant-free hydrothermal process combined with a further heat treatment [42]. The Zn(AC)_2_·2H_2_O was used in the study to synthesise the flower-like ZnO structures, a certain amount of which was dissolved in a solution of NaOH, ethanol and deionized water. The obtained precursor was kept at 150 °C for 24 h. Before decorating with PdO nanoparticles, the flower-like ZnO structures were treated by an annealing process. The annealed ZnO nanoflowers were then dispersed in methanol solvent dissolving PdCl_2_, and the collected products were calcined at 350 °C for 1 h to obtain the PdO-modified ZnO structures. The decorated flower-like ZnO was reported to show a gas sensor response of 35.4 to 100 ppm ethanol at 320 °C (see Figure 4e), which was much higher than that of the pure ZnO (~10 as shown in Figure 4f). Moreover, the composite presented a shorter recovery time of 7 s than that of the ZnO (14 s). ZnO/Co_3_O_4_ composite nanoparticles [64] and Al-doped ZnO/CuO nanocomposites [65] were reported to be sensitive to NO_2_ and ammonia, respectively.

Gao et al. synthesised CuO nanoparticles-decorated MoO_3_ nanorods through a hydrothermal process combined with an annealing process [66]. In the first step, MoO_3_ nanorods were prepared with the raw material (NH_4_)_6_Mo_7_O_24_·4H_2_O. Then, the obtained MoO_3_ nanorods were dispersed in a solution of anhydrous ethanol and copper nitrate under high intensity ultrasonication. The final collected samples were annealed at 550 °C for 2 h. The CuO nanoparticles-decorated MoO_3_ nanorods showed a higher H_2_S sensor response of 272 at 270 °C compared with that of pure MoO_3_, which was mainly attributed to the formation of n-p heterojunctions in the sensing material as reported in their article.

Nano-coaxial Co_3_O_4_/TiO_2_ heterojunctions were successfully assembled through a typical two-step process by Yang et al. [44]. The authors firstly synthesised uniform TiO_2_ nanotubular arrays via anodic oxidation of a Ti plate which were then decorated with Co_3_O_4_ nanoparticles by a hydrothermal process at 120 °C for 5 h. The sensor based on the nano-coaxial Co_3_O_4_/TiO_2_ heterojunctions showed an enhanced sensor response of 40 to 100 ppm ethanol at 260 °C with a short response/recovery time of 1.4 s/7.2 s. The SnO_2_-Co_3_O_4_ composite nanofibres were prepared through electrospinning combined with annealing with the working voltage of 15 kV [67]. The PdO nanoparticle-decorated WO_3_ nanorods were also reported to be synthesised via a modified precipitation process combined with annealing at 300 °C for 2 h [68]. The SnO_2_-Co_3_O_4_ composite nanofibres and the PdO nanoparticle-decorated WO_3_ nanorods were found to be sensitive to 10 ppm C_6_H_6_ at 350 °C and 3.0 vol% of H_2_ at 25 °C with enhanced sensor responses of 20 and 80.4, respectively.

CuO/ZnO heterostructural nanorods were prepared by Cao et al. via a combination of hydrothermal and wet-chemical processes [69]. The CuO nanoparticles-decorated ZnO nanorods array showed a sensor response of ~8 to 50 ppm towards triethylamine at a relatively low working temperature of 40 °C (higher than that of the bare ZnO nanorods of ~2.4), and a response time of 5 s (significantly shorter than that of the pure ZnO nanorods of ~11 s). Improved gas sensing performances were also observed in sensing materials composed of CuO-decorated SnO_2_ nanowires [70], CuO nanoparticles-decorated ZnO flowers [71], flower-like p-CuO/n-ZnO nanorods [72], NiO@ZnO heterostructured nanotubes [73], n-ZnO/p-NiO heterostructured nanofibres [74] and Co_3_O_4_ decorated flower-like SnO_2_ nanorods [75]. The various methods used to assemble the n-p heterojunctions and their gas sensing properties are provided in Table S2.

Besides the nanocomposites discussed above, sensors based on the n-n or n-p heterojunctions have also been assembled to enhance the gas sensing performances of metal oxides. TiO_2_ composited with ZnO, MoS_2_, MoO_3_, V_2_O_5_ and WO_3_ have been designed and successfully established, exhibiting improved gas sensing performances towards ethanol, NO_2_, alcohol and ammonia [33,55,76,77,78]. For example, the ZnO-decorated TiO_2_ nanotube layer (prepared by anodic oxidation combined with atomic layer deposition) [76], TiO_2_/V_2_O_5_ branched nanoheterostructures (synthesised by an electrospinning process followed by an annealing treatment) [33] and a TiO_2_-WO_3_ composite (obtained via plasma spraying technology using mixed feedstock suspensions) [77] have each exhibited promising gas sensing performances to 1170 ppm ethanol, 100 ppm ethanol and 100 ppm NO_2_, respectively. *α*-Fe_2_O_3_ composited with SnO_2_, In_2_O_3_ and CdO have also been successfully synthesised through hydrothermal, carbon sphere template and co-precipitating processes, enabling excellent gas sensitivity towards acetone, TMA and CO, respectively [37,79,80]. In_2_O_3_ composited with WO_3_, Fe_2_O_3_, TiO_2_ and SnO_2_ were also synthesised to assemble the high-performance gas sensors [81,82,83,84]. A series of In_2_O_3_-WO_3_ nanofibres were prepared via an electrostatic spinning technology, which was reported to show an enhanced gas sensing performance to acetone with the n-n semiconductor heterojunctions formed at the interface between WO_3_ and the In_2_O_3_ [81]. The sensor based on mixed Fe_2_O_3_-In_2_O_3_ nanotubes was also reported to show a high gas sensor response of ~33 towards 100 ppm of formaldehyde at 250 °C [82]. TiO_2_ nanoparticle-functionalised In_2_O_3_ nanowires [83], SnO_2_/In_2_O_3_ composite hetero-nanofibres [84], an octahedral-like ZnO/CuO composite [85] and a nanoporous SnO_2_@TiO_2_ heterostructure [86] were reported to show enhanced gas sensing properties towards acetone, formaldehyde and H_2_S, respectively.

Based on the research discussed above, it is clear that the establishment of n-n or n-p heterojunctions can effectively improve the gas sensing properties of n-type metal oxides. Typically, n-type metal oxides are decorated with zero-dimensional nanoparticles and two-dimensional nanosheets, the concentrations of which have significant effects on the performance of the main n-type phase [67,87,88,89]. More specifically, the gas sensing performance of the main phase in a sensing material improves with increasing concentration of the second phase up to an optimal value, which can be attributed to the increase in the specific surface area of the composite. However, the sensing property of the main phase always degenerates when the content of the second phase is further elevated. The interconnection of the second phase and decrease in the effective surface area was reported to be the two main factors causing a weakened sensor response of the composite. However, most of the reported sensors based on the n-n or n-p heterojunctions always worked at temperatures above 100 °C. We also found there are no clear strategies to indicate which material should be chosen to enhance the gas response of a certain n-type metal oxide, which may be paid more attention in the future by researchers.

### 2.2. Improved Gas Sensing Properties of p-n or p-p Junctions

Sensors based on the heterojunctions with p-n or p-p structures were also reported to exhibit promising gas sensing properties with high sensor responses and short response/recovery times. Similar to the formation of n-n or n-p heterojunctions, p-n and p-p junctions are also built with p-type metal oxides (as the main phase) decorated or coated with n-type or p-type metal oxides (as the second phase). The different Fermi levels of the metal oxides in the constructed p-n or p-p junctions induce the formation of a thick accumulation layer and thin depletion layer in the sensing composite. The modulation of the thickness of the accumulation layer (acting as the conductive channel of the carriers) significantly influences the conductivity of the sensors, further resulting in improved gas sensing properties of the p-n or p-p junctions.

#### 2.2.1. Gas Sensors Based on p-n Junctions

**Figure 5:**
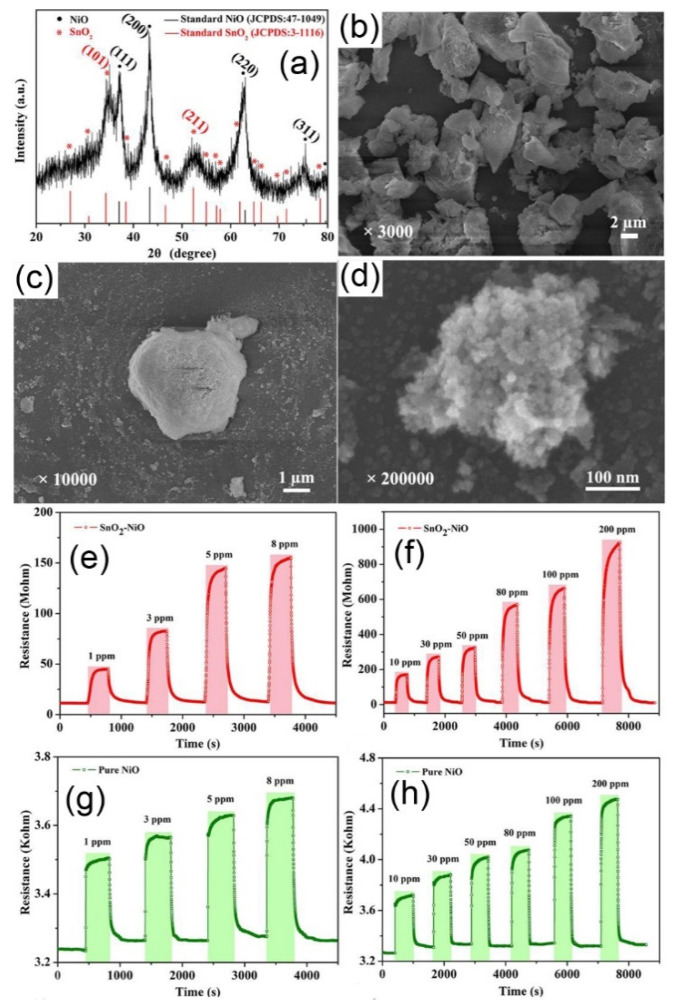
The XRD pattern (**a**) and the SEM images (**b**–**d**) of SnO_2_ nanoparticles-modified NiO nanostructure (p-n junctions), the dynamic sensing performances of the pure NiO and the SnO_2_ nanoparticles-modified NiO nanostructures towards 1–200 ppm toluene at 250 °C (**c**,**e**–**h**). Copied with permission [91]. Copyright 2018, Elsevier.

The p-n heterojunctions in sensing materials have been reported to be effective in improving the gas sensing properties of metal oxides. For example, the SnO-SnO_2_ composite (p-n heterojunction) was successfully prepared by a facile two-step method with the raw materials of SnCl_2_·2H_2_O, NaOH and CTAB at 140 °C for 5 h. Black SnO nanopowders were synthesised via a hydrothermal method at 140 °C for 5 h, and the obtained sample was then treated with an annealing process at a high temperature of 300–500 °C in air atmosphere to obtain the SnO–SnO_2_ composite. The sensor response of the SnO-SnO_2_ composite was 2.5 towards 200 ppm NO_2_ at room temperature, significantly higher than that of pure SnO_2_ (1.27) or bare SnO (1.1) [90]. The hydrothermal method was also applied to synthesise SnO_2_-decorated NiO nanostructures (see Figure 5) with the raw sources of NiCl_2_·6H_2_O and SnCl_4_·5H_2_O at 160 °C for 12 h [91]. It is worth noting that NiO was modified with SnO_2_ nanoparticles through a one-step process without any catalysts. The SnO_2_-decorated NiO nanostructure was reported to show enhanced gas sensor responses to 1–200 ppm toluene (see Figure 5e,f). The calculated sensor response of the composite was measured to be 66.2 to 100 ppm toluene at 250 °C, more than 50 times higher than that of the pure NiO nanospheres (1.3). Moreover, the detection limit of this sensor was reported to be as low as 10 ppb toluene with a promising sensor response of 1.2.

Novel TiO_2_-decorated Co_3_O_4_ acicular nanowire arrays were also successfully synthesised by Li et al. with a hydrogen thermal method combined with pulsed laser deposition. The s acicular nanowire arrays modified with TiO_2_ nanoparticles were found to present a high sensor response of 65 to 100 ppm ethanol at 160 °C, much higher than that of the pure Co_3_O_4_ nanowires (~25) [92]. In_2_O_3_-decorated CuO nanowires were also prepared through thermal oxidation of Cu meshes followed by the deposition of amorphous indium hydroxide from In(AC)_3_ solution in ammonia [93]. The decorated CuO nanowires showed a shorter response time of 12 s to CO than that of the pure CuO nanowires (25 s). The novel rod-like α-Fe_2_O_3_/NiO heterojunction nanocomposites were synthesised with a one-step hydrothermal method, exhibiting an enhanced sensor response of 290 to 100 ppm acetone at 280 °C with a response time or a recovery time being 28 s or 40 s, respectively [94].

Additionally, CuO composited with TiO_2_ [95] and SnO_2_ [35] were constructed to investigate their improved gas-sensing properties. The nanofibres composed of SnO_2_-CuO heterojunctions have been reported to be successfully synthesised by an electrospinning process and exhibited an improved sensor response of ~95 compared with that of the pure CuO (<10) [35]. Co_3_O_4_ composited with In_2_O_3_ [96], SiO_2_ [97] and TiO_2_ [98] were also successfully prepared via hydrothermal, thermal conversion and facile nanoscale coordination polymer routes, respectively, which showed better gas sensing properties than those of pure Co_3_O_4_. The reported sensors based on p-n heterojunctions and their gas sensing performances are listed in Table S3.

#### 2.2.2. Gas Sensors Based on p-p Junctions

The p-p heterojunctions have been found to enhance the gas sensing performance of metal oxides. Li et al. prepared NiO@CuO nanocomposites (a p-p junction) via a facile reflux and hydrothermal process [99]. In their work, the Ni(OH)_2_ was firstly synthesised with the raw material of nickel nitrate hexahydrate through a hydrothermal method at 140 °C for 5 h. Then, the obtained Ni(OH)_2_ and the Cu(CH_3_CO)_2_·H_2_O compounds were added in a solution separately with a certain amount of NaOH added during a reflux process to obtain the Ni(OH)_2_@Cu_2_(OH)_3_NO_3_. The synthesised products were finally treated by a calcination process in air atmosphere at 450 °C for 2 h. The prepared hierarchical flower-like nanostructured NiO-CuO composite exhibited an enhanced gas sensing performance to NO_2_ at room temperature with a higher gas sensor response compared to pure NiO. The response time of the composite to the 100 ppm NO_2_ was measured to be as low as 2 s, much shorter than that of the pure NiO. Moreover, the NiO/NiCr_2_O_4_ nanocomposite was also found to be more effective at detecting xylene than the pure NiO nanoparticles [100].

**Figure 6:**
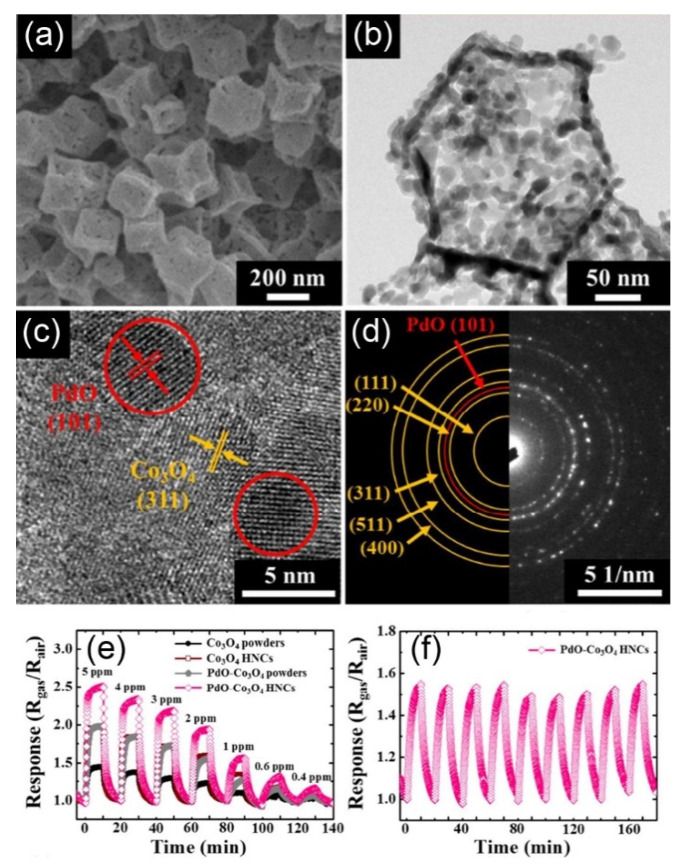
SEM image (**a**), TEM image (**b**), HRTEM (**c**) and SEAD patterns (**d**) of the Co_3_O_4_ hollow nanocages (HNCs) decorated with PdO nanoparticles (p-p junctions), the dynamic sensing behaviours of the sensors based on Co_3_O_4_ powders, pure Co_3_O_4_ hollow nanocages, PdO-Co_3_O_4_ powders and PdO-Co_3_O_4_ HNCs towards 0.4–5 ppm acetone at 350 °C (**e**), the stability of the sensing performance of PdO-Co_3_O_4_ HNCs towards 1 ppm acetone (**f**). Copied with permission [101]. Copyright 2017, American Chemical Society.

Co_3_O_4_ hollow nanocages (HNCs) decorated with PdO nanoparticles (see Figure 6a–d) were successfully assembled by the infiltration of metal precursors combined with a subsequent reduction process [101]. The gas sensing performance of the pure Co_3_O_4_ hollow nanocages was significantly improved when composited with PdO nanoparticles (PdO-Co_3_O_4_ HNCs), with the sensor response measured to be 2.51 towards 5 ppm acetone at 350 °C (see Figure 6e), which was higher than that of the Co_3_O_4_ powders (1.96), Co_3_O_4_ HNCs (1.45) or PdO-Co_3_O_4_ powders (1.98). Moreover, the PdO-Co_3_O_4_ HNCs also exhibited outstanding stability to 1 ppm acetone, which is shown in Figure 6f.

Lee et al. prepared TeO_2_/CuO core-shell nanorods by a combined method of thermal evaporation and sputter deposition [102]. In the reported study, the Te powders were used as the raw material to synthesise TeO_2_. The TeO_2_ nanorods were prepared on a substrate of p-type Si (100) by thermal evaporation of Te powders at 400 °C in air in a quartz tube furnace. Then, a thin layer of CuO was directly sputtered on the surface of the obtained TeO_2_ nanorods through a radio frequency magnetron sputtering process with a target of CuO. The sensor response of TeO_2_-core/CuO-shell nanorods was found to be 4.25 to 10 ppm NO_2_ at 150 °C, which was over two times higher than that of the pure TeO_2_. However, the relatively low sensor response of the TeO_2_/CuO core-shell nanorods is a drawback that limits their application. Further studies are required to further improve the gas sensing performance of the TeO_2_/CuO core-shell nanorods.

Meanwhile, p-NiFe_2_O_4_ nanoparticle-decorated p-NiO nanosheets were also synthesised with a solvothermal method [103]. The NiO precursor was firstly synthesised after which FeCl_3_·6H_2_O was added to prepare NiO nanosheets decorated with NiFe_2_O_4_ nanoparticles. The ratio of Fe/Ni was found to have a significant effect on the gas sensing performance of the decorated NiO nanosheets. The composite with the Fe/Ni-24.9 exhibited the optimal sensing performance to 50 ppm acetone at 280 °C, with a high response of ~23.0. The release of captured electrons back to the sensing material breaks the dynamic carrier balance between p-NiO and p-NiFe_2_O_4_. This resulted in a reduced potential barrier near the surfaces of the heterojunctions and yielded a large variation in resistance, improving the sensor response of the Fe/Ni-24.9 at%. The in situ formation of a second phase (p-NiFe_2_O_4_) on the first phase (p-NiO) was a novel and effective strategy to improve the interaction between the targeted gas and the sensing composite. Similar improvements in CuO-NiO nanotubes with controllable element content of Cu/N developed by a one-pot synthesis was also found, with a sensing capability towards 100 ppm glycol at 110 °C [104]. Based on the studies listed above, the in situ preparation of the second phase required further attention to improve the gas sensing performance of the sensor based on the metal oxide. Other sensors composed with p-p heterojunctions and their gas sensing performances are listed in Table S4.

Other types of heterojunctions based on metal oxides that improve gas sensing performances also exist. Duy et al. assembled n-p-n heterojunctions with the structure of SnO_2_-carbon nanotube-SnO_2_ by the method of CVD combined with spray coating process [105]. The obtained n-p-n heterojunctions showed a high response of 17.9 to 100 ppm NO_2_ at 100 °C. The n-p-n heterostructure of the ZnO-branched SnO_2_ nanowires decorated with Cr_2_O_3_ nanoparticles [106] or the p-n-p heterojunctions of PANI coated CuO-TiO_2_ nanofibres [107] were also reported to exhibit improved gas sensing performance towards hydrogen and ammonia, respectively. However, only a few references report the study of the sensor based on n-p-n or p-n-p heterojunctions. More research should be conducted to systematically investigate the gas sensing properties of metal oxide heterojunctions comprising the n-p-n or the p-n-p structures.

Based on the discussions above, many kinds of metal oxides heterojunctions have been successfully assembled to enhance the gas sensing performance towards various gases. The sensor response of sensors based on heterojunctions was much higher than that of the pure metal oxides and the response time was improved. The n-n, n-p, p-n or p-p (even the n-p-n or p-n-p) heterojunctions can be chosen to be constructed to assemble gas sensors with outstanding properties. We should point out that the enhanced gas sensing mechanisms of certain heterojunctions towards the oxidising or reducing gases need to be clearly discussed and compared to fully understand the role of the heterojunctions. Therefore, in the next section, we review the mechanisms of the improvements in the gas-sensing properties of the metal oxide heterojunctions.

## 3. Enhanced Gas Sensing Mechanisms of the Metal Oxide Heterojunctions

Compared with the pure metal oxides, sensors based on metal oxide heterojunctions show improved gas sensing performances towards the targeted gases. When in contact with each other, the transfer of carriers between the two semiconductor materials is induced due to inconsistent Fermi levels at their interfaces. In the n-n or n-p heterojunctions, the Fermi levels of the two metal oxides will move up or down to an equilibrium state, resulting in the bending of their energy bands and the formation of a potential barrier between them [28]. The gas sensing performances of the studied metal oxides are reported to be mainly attributed to the redox reactions of the adsorbed targeted gases on the surfaces of the sensing materials, which has been widely reported by researchers to explain the gas sensing mechanisms of the assembled sensors [12,108]. The variation in the concentration of the carriers induced by the redox reactions on the surfaces of the composites could be of importance to affect the height of the built-in potential barrier. This process further influences the resistance or conductivity of the sensor based on n-n or n-p heterojunctions according to Equation (1):

Δ*R* ∝ exp{−eΔV_b_/*k_B_T*} (1)

where the Δ*R* is the change of the resistance of the sensor, ΔV_b_ is the reduction of the height of the potential barrier, *k_B_* is the Boltzmann constant and *T* is the temperature [109]. Therefore, little change in the height of a potential barrier would make the resistance of the investigated sensor vary greatly, leading to an improved gas sensing property of the heterojunction [110]. In the case of the p-n or p-p heterojunctions, the interaction between the targeted gases and the sensing materials would also modify the carriers (especially holes) in the sensors, which would further result in the variation of the thickness of the accumulating layer in the heterojunctions, making a more effective modulation in the width of the conduction channel for the carriers. As a result, sensors based on p-n or p-p heterojunctions also show improved gas sensing properties to the reducing or oxidising gases [108]. Moreover, the composites composed of metal oxide heterojunctions always show higher specific surface areas than the pure metal oxides, which was confirmed by BET measurements of the composites. The higher specific surface area enables gas molecules to diffuse more smoothly to the surface and more easily interact with the composite as well as provide more active sites. The size of the pore volume can be increased with a higher specific surface area, facilitating the diffusion of gas molecules into the sensing material and increasing the active surface in internal parts of the composite for gas molecule adsorption. The absorption and the desorption of the gas molecules can also be accelerated during the response and recovery process of the sensor based on metal oxide heterojunctions. Therefore, the high specific surface area forms another positive factor contributing to the comprehensive improvements in the gas sensing performance of the composite [111,112,113,114,115].

Compared with the effect of the specific surface area, it is more complex to study the enhanced gas sensing mechanisms of the heterojunctions in the sensing materials. The role of the heterojunctions in enhanced gas sensing performances should be analysed in detail to fully understand their direct and significant effects on the enhancement of the gas sensing properties of the sensors based on the composites. In the following section, the gas sensing mechanisms of the metal oxides to the common reducing and oxidising gases are discussed, and the effects of various commonly studied heterojunctions on the improved gas sensing properties of the composites are systematically investigated. In order to make the discussions clear, H_2_ (a typical reducing gas) and NO_2_ (a typical oxidising gas) were selected for the discussion of the enhanced gas sensing mechanisms of the metal oxides due to their immense studies in the area of gas sensors.

### 3.1. Enhanced Gas Sensing Mechanisms to Reducing Gases

Gas sensors based on n-n or n-p heterojunctions always exhibit typical n-type sensing performances at relatively low working temperatures towards reducing gases such as H_2_, H_2_S, CO, NH_3_ and ethanol. The widely studied ZnO-based material is taken as an example to more clearly illustrate the sensing mechanism of the n-type metal oxide to a reducing gas. The resistance of ZnO-based sensors has been reported to decrease quickly when H_2_ (ethanol or H_2_S) is introduced onto their surface [116,117,118]. In air, oxygen molecules would spontaneously be adsorbed on the active sites of the surface of the ZnO to form chemisorbed oxygen molecules according to Equation (2). Then, the chemisorbed oxygen molecules can capture electrons from the conductive bands of the ZnO to become the oxygen species (O_2_^−^: <150 °C, O^−^: 150 °C~400 °C and O^2−^: >400 °C) based on Equation (3), which builds a depletion layer in the ZnO surface and a high resistance in air. When H_2_ gas is introduced, the H_2_ molecules will interact with the pre-adsorbed oxygen species to form H_2_O based on Equation (4), releasing electrons back to ZnO. This response process increases the concentration of electrons and decreases the thickness of the depletion layer in ZnO, leading to a decrease in the resistance of the sensor based on ZnO-based materials.

O_2_(*g*) + e^−^ ↔ O_2_(*ad*) (2)

O_2_(*ad*) + e^−^ ↔ O_2_^−^(*ad*) (3)

2H_2_(*g*) + O_2_^−^ (*ad*) = 2H_2_O(*g*) + e^−^ (4)

In contrast, composites made of p-n or p-p heterojunctions show typical p-type sensing performances towards reducing gases. As reported, the resistance of CuO nanowires increased when used to detect hydrogen gas (at working temperatures between 150 °C and 400 °C) [119]. When the CuO nanowires were placed in an air atmosphere, the oxygen molecule could also adsorb on the active sites in the surface of CuO to form adsorbed oxygen species (O^−^) based on Equation (5), releasing holes to CuO and thus increasing the concentration of holes. This forms an accumulation layer in the sensing material, which acts as the conduction channel for carriers in CuO. In an H_2_ atmosphere, hydrogen molecules interact with the adsorbed oxygen molecules according to Equation (6), reducing the concentration of carriers and the thickness of the accumulation layer and induces the formation of a depletion layer on the surface of CuO. Therefore, the resistance of a sensor based on CuO nanowires increases in reducing gas environments [108,119].

O_2_(*g*) ↔ O_2_^−^(*ad*) + h^+^ ↔ 2O^−^(*ad*) + 2h^+^ (5)

2H_2_(*g*) + 2O^−^(*ad*) + 2h^+^ = 2H_2_O(*g*) (6)

**Figure 7:**
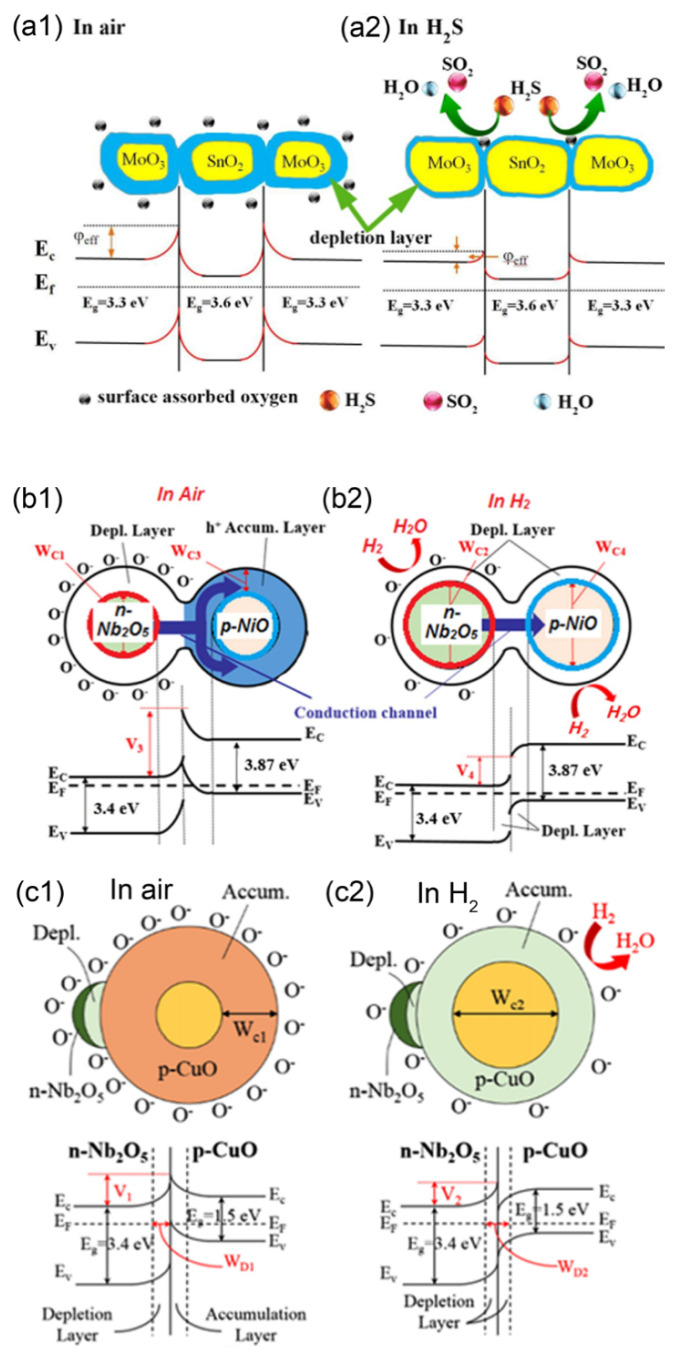
The enhanced gas sensing mechanisms of (**a1**,**a2**) MoO_3_/SnO_2_ nanoflakes (n-n heterojunction) (Copied with permission [120]. Copyright 2019, American Chemical Society), (**b1**,**b2**) NiO-Nb_2_O_5_ composite nanoparticles (n-p heterojunction) (Copied with permission [123]. Copyright 2017, Elsevier) and (**c1**,**c2**) Nb_2_O_5_ nanoparticle-decorated CuO nanorods (p-n heterojunction) (Copied with permission [119]. Copyright 2017, Springer Nature) to reducing gases.

The synthesis of TiO_2_ nanotubes decorated with SnO_2_ nanoparticles and their H_2_ sensing performance has been reported [89] and is selected to analyse the important role of the typical n-n heterojunction in improving the sensing performance of the sensor towards reducing gases. The results showed that the H_2_ sensing property of the TiO_2_-based composite was highly improved with the help of the heterojunction between TiO_2_ and SnO_2_. It was reported that the Fermi level of TiO_2_ was higher than that of SnO_2_, resulting in the electron transfer to SnO_2_ from TiO_2_ until achieving the equilibrium states of their Fermi levels. This would make a thick depletion layer formed at the interface between TiO_2_ and SnO_2_ and induce a high potential barrier built in air due to the adsorption of oxygen molecules. The potential barrier always acts as the obstacle to the transportation of electrons in the sensing materials, resulting in the high resistance of the composites. The accumulation of electrons in the SnO_2_ side would induce more oxygen molecules adsorbed onto the surface of the composite. When hydrogen gas is introduced, the hydrogen gas interacts with the adsorbed oxygen species on the surfaces of TiO_2_ and SnO_2_ immediately and releases electrons back to the sensing materials. The released electrons would decrease the thickness of the depletion layers between TiO_2_ and SnO_2_, further resulting in the decrease in the height of the potential barrier. This process would increase the conductivity of the sensor and significantly enhance the H_2_ sensing performance of the composite. The porous MoO_3_/SnO_2_ nanoflakes with n-n junctions was also reported to show an improved gas sensing property with a higher gas sensor response being 43.5 towards 10 ppm H_2_S at 115 °C compared with that of the pure SnO_2_, which could also be attributed to the reasons mentioned previously (see Figure 7a1,a2) [120]. Moreover, the improvement in the H_2_S or xylene sensing performance of TiO_2_-decorated *α*-Fe_2_O_3_ nanorods [121] or Fe_2_O_3_ nanoparticles-decorated MoO_3_ nanobelts [122] could also be explained by the enhanced gas sensing mechanism above.

In the case of sensors based on n-p heterojunctions, the NiO-decorated Nb_2_O_5_ nanocomposites have been reported to exhibit a significant improvement in the H_2_ gas sensing performance compared with that of the pure Nb_2_O_5_ nanoparticles [123]. When the NiO nanoparticles are loaded onto the surface of the Nb_2_O_5_ nanoparticles, the electrons diffuse to the Nb_2_O_5_ and the holes move toward the NiO, causing the Fermi levels of the two different metal oxides to reach an equilibrium state. In air, the adsorption of the oxygen molecules on the surfaces of the NiO and the Nb_2_O_5_ also results in the formation of an accumulation layer of holes in the NiO side and a depletion layer in the Nb_2_O_5_ side. This causes the energy bands of NiO to bend upwards, increasing the potential barrier at the interfaces in the region of heterojunctions. When the NiO-decorated Nb_2_O_5_ nanocomposites is exposed to H_2_, the interaction with H_2_ and adsorbed oxygen species releases electrons to Nb_2_O_5_ but captures the holes in the NiO. This process induces the formation of a depletion layer between NiO and Nb_2_O_5_ and makes the energy bands of NiO bend downwards, dramatically decreasing the height of the potential barrier at the heterojunction (see Figure 7b1,b2). The NiO nanoparticles have also been reported to be an excellent catalyst to effectively oxidise H_2_, causing reactions between the adsorbed H_2_ and the adsorbed oxygen species to occur more sufficiently and smoothly. As a result, the NiO-decorated Nb_2_O_5_ nanocomposites exhibit an improved gas sensing property to H_2_. The enhanced gas sensing performances of the Co_3_O_4_-decorated WO_3_ nanowires [124], SnO_2_-Co_3_O_4_ composite nanofibres [67], CuO-loaded In_2_O_3_ nanofibres [125], hierarchical SnO/SnO_2_ nanocomposites [126], ZnO nanowire arrays/CuO nanospheres heterostructures [127] and p-NiS/n-In_2_O_3_ heterojunction nanocomposites [34] towards reducing gases can also be attributed to the reasons listed above.

For the p-n junction, Lee et al. reported the sensor based on Nb_2_O_5_ nanoparticles-decorated CuO nanorods to be more sensitive towards hydrogen molecules than the pure CuO nanorods [119]. The higher Fermi level of Nb_2_O_5_ makes the electrons diffuse to the CuO and the holes transfer in an opposite orientation, leading to the bending of energy bands. In air, the adsorption of oxygen molecules captures the electrons from Nb_2_O_5_ but releases the holes to the CuO, resulting in the formation of a thick depletion layer in Nb_2_O_5_ and a thick accumulation layer in the CuO. This leads to the high potential barrier in the composite in air. As reported, the accumulation layer in the CuO can act as a conduction channel for carriers in the sensing material. When exposed to H_2_, the hydrogen molecule can interact with the adsorbed oxygen species on the Nb_2_O_5_ and the CuO, releasing the electrons back to the Nb_2_O_5_ but capturing the holes in the CuO. Effectively, this decreases the thickness of the depletion layer in Nb_2_O_5_ and significantly thins the accumulation layer in CuO with the possible formation of a depletion layer in the CuO, attributed to more oxygen molecules adsorbed on the surface of the CuO due to the formation of heterojunctions. This dramatic decrease in the thickness of the accumulation layer greatly narrows the conduction channel width for carriers, as shown Figure 7(c1,c2). As a result, the Nb_2_O_5_ nanoparticle-decorated CuO nanorods exhibited an improved p-type sensing performance to H_2_. The improved sensing mechanism could also reasonably explain the enhancements in the gas sensing performances of the In_2_O_3_-decorated CuO nanowires [93], SnO_2_-decorated NiO nanostructure [91], hierarchical *α*-Fe_2_O_3_/NiO composites [128], SnO_2_-decorated NiO foam [129] and Cu_x_O-modified ZnO composite [130] with a hollow structure towards H_2_, the toluene and the acetone.

Similarly, in the case of the p-p heterojunction, p-NiO-decorated p-CuO microspheres were prepared through a hydrothermal process and studied the enhanced gas sensing performance of the obtained composite with p-p heterojunctions [131]. The Fermi level of the CuO was higher than that of the NiO, resulting in the transfer of holes from NiO to CuO and the diffusion of electrons to NiO from CuO. As such, accumulation and depletion layers of holes build on the CuO and the NiO sides, respectively. In air atmosphere, the adsorption of the oxygen molecules on the surfaces of CuO and NiO releases holes to the sensing material as previously mentioned. As a result, the thickness of the depletion layer of the holes in the NiO decreases, but the thickness of the accumulation layer of the holes in the CuO increases. The width of the conduction channel increased in the heterojunctions between CuO and NiO, causing a low resistance of the composite. When a reducing gas was introduced on the surface of the composite, the adsorbed oxygen ions reacted with the introduced gas molecules, capturing the holes from CuO and NiO. This process decreases the concentration of the carriers in the sensing materials, further increasing the thickness of the depletion layer of the holes in NiO and decreasing the thickness of the accumulation layer of holes in CuO. Therefore, the width of the conduction channel increased in the heterojunctions was significantly narrowed, causing an increase in the resistance of the composites. Therefore, the gas sensor response of the p-NiO-decorated p-CuO microspheres was highly improved towards reducing gases. The enhanced gas sensing mechanisms of p-NiO/p-NiCr_2_O_4_ nanocomposites [132] or the Cr_2_O_3_-Co_3_O_4_ nanofibres [133] to xylene or C_2_H_5_OH can also be explained by the above discussions.

Apart from H_2_, there are also a number of references reporting similar improved sensing mechanisms of sensors based on metal oxide heterojunctions towards NH_3_, another widely investigated reducing gas. The work conducted by Shi et al. showed that the sensor response of WO_3_@CoWO_4_ (n-n type) heterojunction nanofibres was over 10 times higher than that of the bare WO_3_ at room temperature [134]. The authors pointed out the formation of a number of heterojunctions for WO_3_ composited with CoWO_4_ in the sensing material. The differences in the Fermi levels of WO_3_ and CoWO_4_ cause band bending and trigger the transfer of electrons and holes between them until an equilibrium in final Fermi levels is reached. In air, oxygen gas can be adsorbed on the surface of the two different sensing materials and capture electrons from their conductive bands to form chemisorbed oxygen ions (O_2_^−^ at room temperature) according to Equation (3). This process results in a wider depletion layer and constructs a higher potential barrier near the surface of the heterojunction in the composite than those in the pure CoWO_4_. NH_3_ molecules could interact with the O_2_^−^ according to the following equation: 4NH_3_(*ad*) + 5O_2_^−^ (*ad*) → 4NO(*g*) + 6H_2_O(*g*) + 5e^−^. Electrons were then released back to the sensing materials of the WO_3_@CoWO_4_ composite, reducing the thickness of the depletion layer and decreasing the height of the potential barrier at the heterojunction. As a result, the sensing performance of WO_3_@CoWO_4_ heterojunction nanofibres towards NH_3_ could be significantly enhanced at room temperature. Additionally, the specific surface area of the composite was higher, allowing more electrons to be transferred from the shallow donor levels of the WO_3_ nanoparticles to CoWO_4_ nanoparticles in the composite, thus enabling the enhanced NH_3_ sensing property of the sensor based on the metal oxide heterojunction. The study of Gong et al. also revealed that the enhanced NH_3_ sensing performance of the flower-like n-ZnO decorated with p-NiO with hierarchical structure was mainly attributed to the formation of the depletion layer and the modulation of the potential barrier height at the surface of the heterojunction [135]. A similar improved sensing mechanism was also reported in the enhanced NH_3_ sensing performance of the sensors based on other heterojunctions, including polyaniline/SrGe_4_O_9_ nanocomposite [136], polyaniline nanograin enchased TiO_2_ fibres [137], SnO_2_@polyaniline nanocomposites [138], V_2_O_5_/CuWO_4_ heterojunctions [139], Fe_2_O_3_-ZnO nanocomposites [49], rGO/WO_3_ nanowire nanocomposites [140], WO_3_@SnO_2_ core-shell nanosheets [141], PANI-CeO_2_ nanocomposite thin films [142], CuPc-loaded ZnO nanorods [143], Co_3_O_4_ nanorod-decorated MoS_2_ nanosheets [144], SnO_2_/NiO composite nanowebs [145], bilayer SnO_2_-WO_3_ nanofilms [146], Cu_2_O nanoparticles decorated with p-type MoS_2_ nanosheets [147], TiO_2_ and NiO nanostructured bilayer thin films [148] and mesoporous In_2_O_3_@CuO multijunction nanofibres [149].

### 3.2. Improved Gas Sensing Mechanism towards Oxidising Gases

In contrast to the sensing behaviour of n-n or n-p heterojunctions towards reducing gases, sensors based on n-n or the n-p heterojunctions were reported to exhibit a typical p-type sensing performance towards the oxidising gases. Many researchers have studied the oxidising gas (such as NO_2_) sensing performance of heterojunctions based on n-type metal oxides at working temperatures within the range of 150 °C to 400 °C. The ZnO nanoparticles exhibited a typical p-type sensing performance towards 0.3–10 ppm NO_2_ at the working temperature of 250 °C, with the resistance of the sensor increasing quickly when exposed to an NO_2_ gas atmosphere [150]. In air, oxygen molecules can adsorb onto the active sites of the surface of the nanostructured ZnO according to Equation (7), capturing electrons from the conductive bands of ZnO. This process causes a decrease in carriers in ZnO and the formation of a depletion layer on the surface of ZnO. When exposed to an NO_2_ atmosphere, the NO_2_ molecules can interact with the ZnO directly and with adsorbed O^−^ on the ZnO according to Equations (8) and (9), respectively. Generally, NO_2_ can be adsorbed onto the active sites of the ZnO surface based on Equation (8), capturing the electrons from the ZnO to form NO_2_^−^. The NO_2_^−^ can then further react with adsorbed oxygen species following Equation (9), grabbing electrons from ZnO. These sensing processes decrease the concentration of the carriers in the ZnO and increase the thickness of the depletion layer in the surface of the ZnO, resulting in a significant increase in the resistance of ZnO and the p-type sensing performance to NO_2_ gas.

1/2O_2_(*g*) + e^−^ ↔ O^−^(*ad*) (7)

NO_2_(*g*) + e^−^ ↔ NO_2_^−^(*ad*) (8)

NO_2_^−^(*ad*) + O^−^(*ad*) + 2e^−^ ↔ NO(*g*) + 2O_2_^−^ (9)

In addition, sensors based on p-n or p-p heterojunctions have been found to show typical n-type sensing performances towards oxidising gases. The resistances of the sensors assembled by heterojunctions based on p-type metal oxides decrease rapidly when exposed to oxidising gases at the working temperatures of approximately 200 °C. Taking the sensor based on Co_3_O_4_ as an example, oxygen molecules can be adsorbed onto the active sites on the surface of Co_3_O_4_ according to Equation (5), releasing holes to Co_3_O_4_ and resulting in the formation of chemisorbed oxygen species (mainly O^−^). This process can also induce the establishment of an accumulation layer in the surface of Co_3_O_4_. In an NO_2_ atmosphere, NO_2_ molecules have also been reported to adsorb onto the active sites of a Co_3_O_4_ surface based on Equation (10), releasing holes to the sensing materials and forming NO_2_^−^. The adsorbed NO_2_^−^ can then interact with the adsorbed oxygen species according to Equation (11), releasing more holes to Co_3_O_4_. These processes make the accumulating layer thicker on the surface of Co_3_O_4_ and cause the resistance of the sensor to decrease, leading to an n-type sensing performance of the Co_3_O_4_-based sensor towards NO_2_ [64].

NO_2_(*g*) ↔ NO_2_^−^(*ad*) + h^+^ (10)

NO_2_^−^(*ad*) + O^−^(*ad*) ↔ NO(*g*) + 2O_2_^−^(*ad*) + 2h^+^ (11)

**Figure 8:**
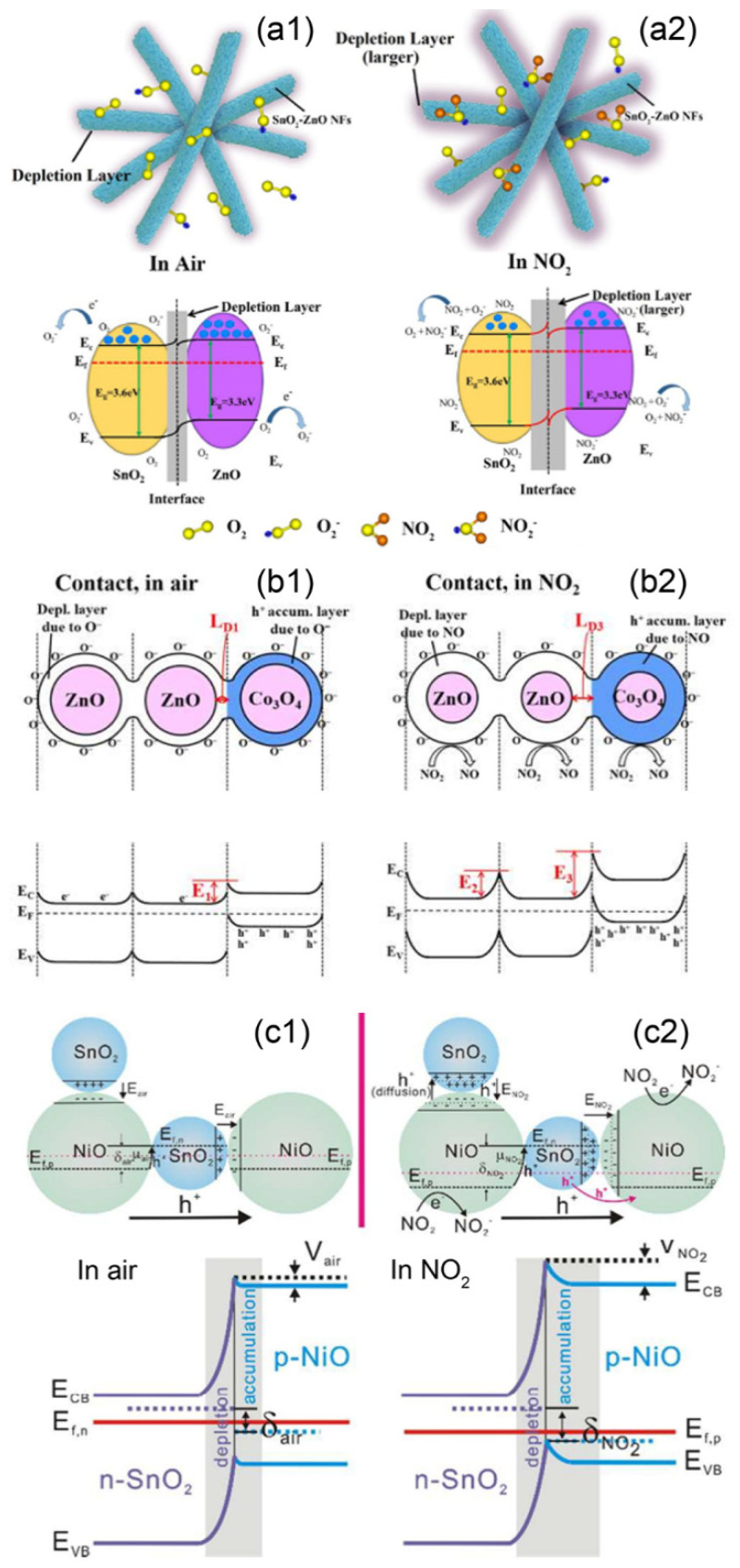
The enhanced gas sensing mechanisms of (**a1**,**a2**) the hollow nanofibres of ZnO-SnO_2_ (n-n heterojunction) (Copied with permission [151]. Copyright 2018, Elsevier.), (**b1**,**b2**) the ZnO/Co_3_O_4_ composite nanoparticle (n-p heterojunction) (Copied with permission [64]. Copyright 2016, Elsevier.) and (**c1**,**c2**) NiO-SnO_2_ nanocomposites (p-n heterojunction) (Copied with permission [154]. Copyright 2016, Royal Society of Chemistry.) towards oxidising gases.

For the sensor based on an n-n heterojunction, the study of the gas sensing properties of ZnO-SnO_2_ hollow nanofibres showed that the composites exhibit a much higher sensor response towards NO_2_ than pure SnO_2_ [151]. In the composite, the Fermi level of the SnO_2_ is higher than that of the ZnO. The lower Fermi level of the ZnO can thus lead to the transfer of the holes from ZnO to SnO_2_ and the diffusion of electrons to ZnO from SnO_2_ until their Fermi levels reach an equilibrium state. This process can then form a thick accumulation layer on the ZnO side and a thin depletion layer on the SnO_2_ side. In air atmosphere, oxygen molecules can adsorb onto the surface of SnO_2_, which would capture electrons from SnO_2_ and increase the thickness of the built-in depletion layer. The accumulation layer of the electrons in ZnO can cause more molecules to absorb onto its surface in air, capturing electrons and significantly decreasing the thickness of the established accumulation layer and even lead to the formation of a thin depletion layer (see Figure 8(a1)). In an NO_2_ atmosphere, adsorbed NO_2_ molecules on the surfaces of the metal oxides and the reaction between NO_2_ and adsorbed oxygen molecules further capture electrons in ZnO and SnO_2_, significantly increase in the depletion layer at the interfaces between ZnO and SnO_2_ (see Figure 8(a2)). As a result, the height of the potential barrier increases greatly, making the ZnO-SnO_2_ hollow nanofibres exhibit an improved NO_2_ gas sensing property. The sensors based on ZnO nanorods decorated with TiO_2_ nanoparticles [61], Bi_2_O_3_-branched SnO_2_ nanowires [112], In_2_O_3_-composited SnO_2_ nanorods [152] and SnO_2_-core/ZnO-shell nanowires [153] also exhibited improved NO_2_ gas sensing performances according to the mechanism discussed above.

In the case of the n-p junction, Co_3_O_4_-decorated ZnO nanoparticles have been established by Lee et al. and showed a significant enhancement in the NO_2_ gas sensing performance [64]. The Fermi level of ZnO is higher than that of Co_3_O_4_, inducing the transfer of carriers between them and the formation of a depletion layer at the heterojunction. In air, the adsorption of the oxygen molecules on the surfaces of ZnO and Co_3_O_4_ capture the electrons from ZnO and release holes to Co_3_O_4_. This leads to the building of a depletion layer on the ZnO side and an accumulation layer on the Co_3_O_4_ side as well as a significant bending in their energy bands (see Figure 8(b1)). As a result, a potential barrier is formed at the interfaces between ZnO and Co_3_O_4_, resulting in a higher resistance of the sensor based on the composites than that of pure ZnO. In an NO_2_ atmosphere, the adsorption of NO_2_ molecules on the surfaces of ZnO and Co_3_O_4_ can lead to the capture of electrons from ZnO but the release of the holes to Co_3_O_4_. The variation in the carriers in ZnO and Co_3_O_4_ causes both the depletion layer in ZnO and the accumulation layer in Co_3_O_4_ to become thicker. This sensing process increases the height of the potential barrier in the heterojunctions and significantly increases the resistance of the Co_3_O_4_-decorated ZnO composite (see Figure 8(b1,b2)). Meanwhile, the catalytic property of Co_3_O_4_ to NO_2_ also acts as a positive factor for the improved NO_2_ gas sensing performance of the composite. Oxygen molecules are reported to be more easily adsorbed onto the surface of p-type metal oxides, which is another reason for the improved NO_2_ gas sensing performance of the Co_3_O_4_-decorated ZnO nanoparticles. The improvements in the NO_2_ gas sensing properties of the SnO-SnO_2_ nanocomposites [90], CuO-decorated ZnO nanowires [155], TeO_2_/SnO_2_ brush- nanowires [156] and ultra-long ZnO@Bi_2_O_3_ heterojunction nanorods [157] can also be attributed to the reasons listed above.

Sensors based on the p-n heterojunctions have also been reported to be effective at detecting oxidising gases. For example, NiO-SnO_2_ nanocomposites (p-n junctions) were found to exhibit an improved gas sensing performance towards NO_2_ compared with pure NiO [154]. The Fermi level of the n-type SnO_2_ was found to be higher than that of the p-type NiO. The electrons would be transferred from SnO_2_ to the NiO, and the holes would diffuse from the NiO to the SnO_2_. In an air atmosphere, the adsorption of oxygen molecules would capture electrons in the SnO_2_ and release the holes to NiO, resulting the formation of a thin depletion layer in SnO_2_ and an accumulation layer in NiO. A potential barrier is then established between NiO and SnO_2_, and the carriers in the sensing materials are mainly transported through the accumulation layer. In an NO_2_ atmosphere, the adsorption of NO_2_ molecules on the surface of NiO results in more holes being released to NiO, further increasing the thickness of the accumulation layer in the NiO layer. The adsorption of NO_2_ molecules on the surface of SnO_2_ would allow more electrons to be grabbed from the SnO_2_, further increasing the thickness of the depletion layer in SnO_2_. Moreover, NO_2_ can be adsorbed on SnO_2_ more easily due to its higher electron concentration. There would be more NO_2_ molecules adsorbed on the NiO-SnO_2_ nanocomposites, further resulting in the great modulation in the accumulation layer of the heterojunction nanocomposites. The increase in the thickness of the accumulation layer in NiO would widen the conduction channel for the carriers, which would result in a significant decrease in the resistance of the sensing material (see Figure 8(c1,c2)). Therefore, the sensors based on NiO-SnO_2_ nanocomposites exhibit a higher sensor response to NO_2_ than that of bare NiO.

For the p-p heterojunction, the NO_2_ gas sensing performance of sensors based on p-type NiO nanosheets could be successfully improved through modifying them with the p-type CuO nanoparticles [158]. In the CuO-decorated NiO nanosheets, the differences in Fermi levels of CuO and NiO lead to the transfer of carriers between the two, resulting in the formation of a hole depletion layer and a hole accumulation layer between their interfaces. In air, the adsorption of oxygen molecules on the surfaces of CuO and NiO can release holes to the sensing materials, leading to the increase in the thickness of the accumulation layer at the interfaces between CuO and NiO. When NO_2_ is introduced and interacts with the sensing material, more holes are released to CuO and NiO. Moreover, more NO_2_ molecules become adsorbed on the sensing material due to the accumulation of holes in the composite and its higher specific surface area. This sensing process would more effectively increase the carriers (holes) in the composites and widen the accumulation layer between CuO and NiO. As a result, the resistance of the p-p heterojunctions significantly decreased and the CuO-decorated NiO nanosheets presented an enhanced NO_2_ gas sensing performance. The enhanced NO_2_ sensing mechanism discussed above can also be applied to explain the improved NO_2_ sensing properties of the sensors based on CuO-decorated TeO_2_ nanorods [102] and vertically aligned Cu_3_Mo_2_O_9_ micro/nanorods on a CuO layer (Cu_3_Mo_2_O_9_@CuO nanorods) [159].

The discussions of the enhanced gas sensing mechanisms of the n-n, n-p, p-n and p-p heterojunctions reveal that modulations of the height of the potential barriers and the thickness of the accumulation layer in the heterojunctions are responsible for the improvements of the gas sensing performances of the nanocomposites. The different Fermi levels of the metal oxides induce band bending in the heterojunctions, leading to the formation of potential barriers and accumulation layers in n-type and p-type heterojunctions, respectively. The interactions between the targeted gases and the sensing materials cause variations in the height of the potential barriers in n-type heterojunctions (n-n or n-p heterojunctions) and the thickness of the accumulation layer in p-type heterojunctions (p-n or p-p heterojunctions), inducing enhancements of the sensing performance of the composites.

## 4. Conclusions

The gas sensing performances of metal oxides have been successfully improved through assembling heterojunctions in sensing materials. The heterojunctions are usually constructed via combined methods of electrospinning, thermal oxidation, ALD, PLD, hydrothermal process and CVD. The sensor response, response time or recovery time based on n-n, n-p, p-n or p-p heterojunctions can be effectively enhanced. Modulations in the built-in heterojunctions are mainly responsible for the enhanced gas sensing performances of the sensors based on n-n or n-p junctions. The improvement in the gas sensing behaviours of the sensors based on p-n or p-p heterojunctions can be attributed to variations in the thicknesses of the accumulation layers in the junctions. n-type or p-type nanostructured metal oxides with different morphologies can be selected to assemble heterojunctions and their concentrations can modified, indicating that more interesting gas sensors based on nanostructured metal oxide heterojunctions might be explored.

## Supplementary Materials

The following are available online at https://www.mdpi.com/article/10.3390/nano11041026/s1, Table S1: The assembled strategies of n-n heterojunctions and their gas sensing performances. Table S2: The assembled strategies of n-p heterojunctions and their gas sensing performances. Table S3: The assembled strategies of p-n heterojunctions and their gas sensing performances. Table S4: The assembled strategies of p-p heterojunctions and their gas sensing performances.
