# Supplementary material for: Metal Oxide Based Heterojunctions for Gas Sensors: A Review
Source: Nanomaterials (Basel). 2021 Apr 17;11(4):1026. doi: 10.3390/nano11041026 (PMC8073732; doi:10.3390/nano11041026)
Supplement: Supplementary file 1 [file nanomaterials-11-01026-s001.zip › nanomaterials-1168608-SI.pdf]

nanomaterials

Review

Metal Oxide Based Heterojunctions for Gas Sensors: A Review

Shulin Yang [1,2], Gui Lei [1,2], Huoxi Xu [1,*], Zhigao Lan [1], Zhao Wang [2] and Haoshuang Gu [1,2,*]

1 Hubei Key Laboratory for Processing and Application of Catalytic Materials, School of Physics and Electronic Information, , Huanggang Normal University, Huanggang 438000, China; yangsl@hgnu.edu.cn (S.Y.); leig@hgnu.edu.cn (G.L.); lanzhg@hgnu.edu.cn (Z.L.)
2 Hubei Key Laboratory of Ferro & Piezoelectric Materials and Devices, Faculty of Physics and Electronic Sciences, Hubei University, Wuhan 430062, China, wangzhao@hubu.edu.cn
* Correspondence: author: xuhuoxi@hgnu.edu.cn (H.X.); guhsh@hubu.edu.cn (H.G.)

Table S1. The assembled strategies of n-n heterojunctions and their gas sensing performances.

Sensing composites	Type of junctions	Synthesis method	Working temperature (°C)	Sensing gas/conc. (ppm)	Sensor response (R_a/R_g) or (R_g/R_a)[a]	Response time (s)	Recovery time (s)	References
α-Fe_2O_3 nanoparticles decorated ZnO nanoparticles	n-n	Sol-gel and spin-coating method	Room temperature	NH_3/0.4	10000	20	20	[1]
SnO_2 nanoparticles modified ZnO nanorods	n-n	Wet chemical method	20	NO_2/200	2.5	720	840	[2]
SnO_2-ZnO composite Nanofibers	n-n	Electrospinning	300	H_2/10	168.6	172	530	[3]
CeO_2 decorated ZnO nanosheets	n-n	Hydrothermal method combination with wet impregnation method	310	Ethanol/100	90	20	4	[4]
Sb-doped SnO_2 covered ZnO nano-heterojunction	n-n	Microwave hydrothermal method	NA	NO_2/1	1.95	16	NA[b]	[5]
SnO_2-coated ZnO nanowire	n-n	Solvothermal treatment followed with calcination	240	N-butylamine/200	9.2	< 30	< 55	[6]
TiO_2 nanoparticles decorated ZnO nanorods	n-n	Sol-gel technology followed by a spin coating method	120	NO_2/50	112	14	6	[7]
ZnO tetrapod alloyed with Fe_2O_3 nanoparticles	n-n	Flame transport synthesis	300	Ethanol/200	57.56	65	220	[8]
SnO_2-TiO_2 composite oxide	n-n	Sol-gel method	280	Ethanol/200	62	~ 10	~ 8	[9]
CeO_2-SnO_2 mixed oxide heterostructure	n-n	Hydrothermal method	300	H_2/40	19.23	17	24	[10]
SnO_2 nanoparticles decorated SnS_2	n-n	In-situ high-temperature oxidization	80	NO_2/8	5.3	159	297	[11]
Ultrathin mesoporous ZnO-SnO_2 nanosheets	n-n	Urea decomposition method and subsequently calcination	240	Ethanol/50	80	7	42	[12]
TiO_2 nanoparticles decorated SnO_2 nanosheets	n-n	Hydrothermal process and PLD process	260	Triethylamine/100	52.3	16	22	[13]
Mg-doped TiO_2/SnO_2 nanosheets	n-n	Hydrothermal method combined with and PLD	260	Triethylamine/50	30.4	9	95	[14]
SnO_2-ZnO composites	n-n	Chemical route	90	H_2/10000	~ 16	60	75	[15]
SnO_2 nanoparticles decorated MoS_2 nanoflowers	n-n	Hydrothermal method combined with PLD	300	Triethylamine/100	68.7	12	84	[16]
Fe_2O_3 nanoparticles decorated SnO_2 nanowires	n-n	VSL combine with hydrothermal process	300	Ethanol/200	57.56	~ 65	~ 200	[17]
Mixed SnO_2/TiO_2 included with carbon nanotubes	n-n	Sol-gel spin-coating method	250	Ethanol/1000	41	NA	NA	[18]
α-MoO_3/TiO_2 core/shell nanorods	n-n	Hydrothermal method	180	Ethanol/10	4.8	< 40	< 40	[19]
Nano-coaxial Co_3O_4/TiO_2 heterojunction	n-n	Anodic oxidation combined with hydrothermal method	260	Ethanol/100	40	1.4	7.2	[20]
Brush-like ZnO-TiO_2 heterojunctions nanofibers	n-n	Electrospinning and hydrothermal process.	320	Ethanol/500	50	5	10	[21]

TiO_2/V_2O_5 branched nanoheterostructures	n-n	Electrospinning process combined with annealing	350	Ethanol/100	24.6	6	7	[22]
MoS_2 decorated TiO_2 nanotube	n-n	Anodization combined hydrothermal process	150	Ethanol/100	14.2	~ 15	~ 15	[23]
SnO_2 decorated TiO_2 nanotubes	n-n	Anodization combined with impregnation method.	250	H_2/1000	1140	2	~ 400	[24]
TiO_2 nanotube arrays by ZnO modification	n-n	Anodization combined with impregnation method.	300	H_2/100	340	22	2000	[25]
SnO_2 nanoparticles decorated TiO_2 nanofibers	n-n	Electrospinning technique	240	Ethanol/100	9.58	8	10	[26]
WO_3 nanorods decorated ZnO nanoplates	n-n	Hydrothermal treatment	250	NH_3/300	24	60	50	[27]
WO_3 nanoclusters-SnO_2 film	n-n	Pulsed laser deposition technique	100	NO_2/10	51000	67	17.05	[28]
SnO_2 nanoparticles modified WO_3 nanolamella	n-n	Acidification method combined with calcination	200	NO_2/0.2	370	NA	NA	[29]
MoO_3-ZnO core-shell nanorods	n-n	Hydrothermal method combined with ALD	350	Ethanol/200	7.62	44.88	119.87	[30]
MoS_2-MoO_3 hybrid microflowers	n-n	Controlled vapor transport process	25	NO2/10	1.35	19	182	[31]
ZnO nanoparticles deorated	n-n	Hydrothermal method	250	Ethanol/100	18	2.5	5	[32]
MoO_3/ZnO composite	n-n	Hydrothermal method	270	H_2S/100	30	13	29	[33]
MoO_3/Fe_2O_3 composite	n-n	Hydrothermal strategy	206	Xylene/100	6.9	87	190	[34]
MoO_3/V_2O_5 composite	n-n	Spray pyrolysis deposition	200	NO_2/100	1.8	118	1182	[35]
MoO_3/SnO_2 composite	n-n	Wet-chemical method combined with calcination	115	H_2S/10	43.5	22	10	[36]
Fe_2O_3/WO_3 nanocomposites	n-n	Two-step solution-based method	260	Acetone/100	105.8	7	20	[37]
In_2O_3/α-Fe_2O_3 heterostructure nanotubes	n-n	Single-capillary electrospinning method	225	Ethanol/100	24.41	3-5	6-10	[38]
TiO_2 nanoparticles decorated α-Fe_2O_3 nanorods	n-n	Thermal oxidation combined with solvothermal treatment	300	H_2S/200	7.4	~ 150	~ 155	[39]
Fe_2O_3/ZnO core/shell nanorod	n-n	Solution phase controlled hydrolysis method	200	Ethanol/50	6.48	< 20	< 20	[40]
ZnO/Fe_2O_3 heterostructure	n-n	Hydrothermal method combined with ALD	250	H_2S/50	~ 100	19.1	156.5	[41]
ZnO nanoparticles decorated α-Fe_2O_3	n-n	Hydrothermal method	200	Acetone/200	~ 43	19	20	[42]
TiO_2-In_2O_3 composite nanofibers	n-n	Electrospinning method	25	NO_2/0.3	NA	0.1	10.3	[43]
Mixed Fe_2O_3-In_2O_3 nanotubes	n-n	Electrospinning	250	Formaldehyde/100	33	5	25	[44]
Fe_2O_3 nanoparticles modified In_2O_3 nanowires	n-n	Thermal evaporation followed by solvothermal deposition	200	Acetone/500	~ 10	~ 55	~80	[45]
SnO_2-In_2O_3 composite	n-n	Solid-phase reaction method	160	Methanol/100	320.73	32	47	[46]
ZnO-modified In_2O_3 heterojunction	n-n	Hydrothermal method combined	300	HCHO/100	46.8	6	7	[47]

		with ultrasonic reaction						
In_2O_3-core/ZnO-shell nanorod	n-n	Thermal evaporation followed by ALD	300	H_2S/100	1.28	530	500	[48]
Core-Shell In_2O_3/ZnO nanoarray	n-n	Hydrothermal process combined with a wet-chemical method	Room temperature	H_2S/700	925	NA	NA	[49]
SnO_2 hollow spheres decorated with CeO_2 nanoparticles	n-n	Two-step hydrothermal strategy	225	Ethanol/100	37	2	70	[50]
Branch-like α-Fe_2O_3/TiO_2 nanofiber	n-n	Electrospinning technique and hydrothermal process	95	TMA/50	15	75	112	[51]
ZnO-SnO_2 composite	n-n	Hydrothermal method	26	O_3/0.6	12	13	90	[52]
In_2O_3 nanoparticles on ZnO hollow nanotubes	n-n	Hydrothermal method	260	Ethanol/100	68.19	8	10	[53]

[a]In this table, the R_a and the R_g were the resistances of the sensor in the air and the resistances of the sensor in the targeted gases, respectively.[b]The NA in the table means that the related information was not given in the references.

Table S2. The assembled strategies of n-p heterojunctions and their gas sensing performances.

Sensing composites	Type of junctions	Synthesis method	Working temperature (°C)	Sensing gas/conc. (ppm)	Sensor response (R_a/R_g) or (R_g/R_a)[a]	Response time (s)	Recovery time (s)	References
NiO nanoparticles modified ZnO nanorods	n-p	Two-step chemical bath deposition	330	Acetone/100	~ 13	~ 5	~ 10	[54]
NiO/ZnO nanofibers	n-p	Electrospinning	260	TMA/100	892	18	20	[55]
CuO nanoparticles decorated ZnO nanorods	n-p	Solution-based synthesis method	25	NO/10	3.65	100	> 600	[56]
CdO nanoparticles-decorated flower-like ZnO hollow microspheres	n-p	Two-step hydrothermal strategy	250	Ethanol/100	65.5	2	136	[57]
ZnO nanowire arrays/CuO nanospheres heterostructure	n-p	Hydrothermal method	122	Ethanol/80	NA[b]	41	99	[58]
ZnO/CuO composite	n-p	Solvothermal method	40	H_2S/10	23.03	173	> 3000	[59]
PdO-decorated flower-like ZnO structures	n-p	Hydrothermal route combined with heat treatment	320	Ethanol/100	35.4	1	7	[60]
Cr_2O_3-functionalization ZnO nanorods	n-p	Carbothermal synthesis combined with solvothermal process	25	Ethanol/200	10.95	26	110	[61]
CuO nanoparticles decorated SnO_2 nanowires	n-p	Two-step CVD	200	H_2S/1	700	NA	NA	[62]
Co_3O_4 decorated flower-like SnO_2 nanorods	n-p	Hydrothermal method followed by chemical solution method	280	Xylene/100	47.8	98	107	[63]
NiO-SnO_2 composite nanofibers	n-p	Electrospinning	320	H_2/100	~ 14	~ 3	~ 3	[64]
NiO/SnO_2 hollow sphere	n-p	Hydrothermal method followed by PLD process	220	Triethylamine/10	48.6	11	34	[65]
CuO nanoparticles decorate SnO_2 nanorods	n-p	Hydrothermal method	60	H_2S/10	94000	~ 60	NA	[66]
NiO-SnO_2 microflowers	n-p	Hydrothermal route	200	Formaldehyde/100	~ 27.5	20	64	[67]
Bi_2O_3-branched SnO_2 nanowires	n-p	Vapor-liquid-solid method	250	NO_2/2	56.92	~ 195	~ 20	[68]
Co_3O_4/SnO_2 flower-like structures	n-p	One-step hydrothermal technique	175	Trimethylamine/5	9.3	19	29	[69]
CeO_2/TiO_2 core/shell nanorods	n-p	Hydrothermal method	320	Ethanol/1000	5.44	< 45	< 45	[70]
Co_3O_4/TiO_2 nanotube heterostructures	n-p	Anodization combined and cathodic deposition	200	H_2/1000	1.06	~ 720	~ 600	[71]
CuO nanoparticles decorated WO_3 nanoplates	n-p	Hydrothermal method	100	H_2S/5	223	73	450	[72]
NiO-decorated WO_3 nanocomposite	n-p	Hydrothermal process combined with solvothermal method	350	Ethanol/150	~ 80	~ 10	~ 5	[73]
CuO-modified WO_3 thin film	n-p	RF sputtering technique	300	H_2S/10	534	2	~ 1440	[74]
CuO-functionalized WO_3 nanowires	n-p	Thermal evaporation combined with thermal annealing	300	H_2S/100	6.73	~ 60	~ 70	[75]
Cr_2O_3 nanoparticle modified WO_3 nanorods	n-p	Thermal evaporation followed by spin-coating	300	Ethanol/200	5.58	~ 50	~ 60	[76]
WO_3/CuO composites	n-p	Hydrothermal method	80	H_2S/5	105.14	42	3500	[77]
CuO nanoparticles decorated α-MoO_3 nanorods	n-p	Hydrothermal method combined with heating	180	H_2S/50	160.8	~ 20	40	[78]
PdO nanoparticles decorated WO_3 nanoneedle	n-p	Aerosol assisted CVD	200	H_2/500	1500	120	720	[79]
Fe_2O_3/Co_3O_4 composite	n-p	Hydrothermal route	300	Ethanol/100	10.86	1.36	40.25	[80]
p-NiS/n-In_2O_3 heterojunction nanocomposites	n-p	Solid-state grinding and recalcination	300	Ethanol/100	10.8	8	20	[81]

Bi_2O_3 nanoparticles decorated In_2O_3 nanorods	n-p	One-step CVD process	200	Ethanol/200	17.74	23	200	[82]
NiO-In_2O_3 composite nanofibers	n-p	Electrospinning and calcination method	300	Ethanol/100	78	~ 6	60	[83]
SnO_2-Co_3O_4 composite nanofibers	n-p	Electrospinning method	350	C_6H_6/1	18.7	13.58	13.65	[84]
ZnO/Co_3O_4 composite nanoparticle	n-p	Solvothermal route	Room temperature	NO_2/5	15	~ 17.5	~ 32.5	[85]
Porous flower-like CuO/ZnO nanostructures	n-p	Chemical solution method	220	Ethanol/100	25.5	6	42	[86]
CuO/ZnO heterostructured nanorods	n-p	A photochemical method	500	H_2S/50	891	~ 820	~ 20	[87]
CuO-modified SnO_2 film	n-p	Screen printing technique combined with firing process	25	H_2S/1	3672	< 15	~ 240	[88]
CuO nanoparticles modified SnO_2 nanowire	n-p	Chemical vapour deposition	200	H_2S/2	3261	< 180	< 600	[89]
CuO nanoparticle decorated ZnO nanorod	n-p	Two-stage solution process	100	H_2S/100	~ 38	~ 120	~ 120	[90]
CuO nanoparticles decorated ZnO nanoparticles	n-p	Hydrothermal method	200	Alcohol/200	3.3	62	83	[91]
CuO/ZnO heterojunction nanorods	n-p	Hydrothermal process combined with annealing	300	Ethanol/100	98.8	7	9	[92]

[a]In this table, the R_a and the R_g were the resistances of the sensor in the air and the resistances of the sensor in the targeted gases, respectively. [b]The NA in the table means that the related information was not given in the references.

Table S3. The assembled strategies of p-n heterojunctions and their gas sensing performances.

Sensing composites	Type of junctions	Synthesis method	Working temperature (°C)	Sensing gas/conc. (ppm)	Sensor response (R_a/R_g) or (R_g/R_a)[a]	Response time (s)	Recovery time (s)	References
NiO-SnO_2 foam	p-n	Dip-coating process	210	Toluene/10	19.2	9	8	[93]
NiO/ZnO heterojunction diode	p–n	Spin-coating followed by hydrothermal method	300	Toluene/95	20.1	100	60	[94]
α-Fe_2O_3/NiO heterojunction nanorods	p-n	Hydrothermal method	280	Acetone/100	290	28	40	[95]
Hierarchical α-Fe_2O_3/NiO composites	p-n	Hydrothermal method	200	Toluene/100	16.38	1	12	[96]
Cr_2O_3 nanoparticles decorated In_2O_3 nanorods	p-n	Thermal evaporation followed by solvothermal deposition	200	Ethanol/500	10.53	~ 17.5	~ 50	[97]
Fe_2O_3/Co_3O_4 codecorated In_2O_3 nanorod	p-n	Thermal evaporation followed by solvothermal deposition	200	Ethanol/200	35	10	120	[98]
Co_3O_4/ZnO nanocomposites	p-n	Chemical vapor deposition	400	CH_3COCH_3/100	1.2	NA[b]	NA	[99]
Co_3O_4/α-Fe_2O_3 heterostructure nanofibers	p-n	Coaxial electrospinning method	240	Acetone/50	11.7	2	20	[100]
Co_3O_4-SnO_2 hollow hetero-nanostructures	p-n	Galvanic replacement	275	Ethanol/5	13.5	NA	NA	[101]
Hierarchical Fe_3O_4@Co_3O_4 core-shell microspheres	p-n	Hydrothermal method	160	Acetone/100	100	5	15	[102]
Rh_2O_3 clusters decorated WO_3 crystallites	p-n	Wet-chemical method	300	CO/0.9	NA	NA	NA	[103]
Co_3O_4/SnO_2 core-shell nanospheres	p-n	Hydrothermal method	200	NH_3/50	13.5	4	17	[104]
ZnO-Co_3O_4 hollow polyhedrons heterostructure	p-n	Thermal decomposition	200	Ethanol/1000	106	7	236	[105]
ZnO/Co_3O_4 microspheres	p-n	Solvothermal method combined annealing process	275	Ethanol/50	120	5.6	29	[106]
Co_3O_4-TiO_2 composite	p-n	Thermal conversion	115	Xylene/50	113	130	150	[107]
Ultra-thin CuO islands on sputtered SnO_2	p-n	Sputtering followed by thermal evaporation	150	H_2S/20	7400	15	118	[108]
CuO/ZnO thin film heterojunction	p-n	Sol-gel technique	300	H_2/3000	266.5	NA	NA	[109]
CuO@ZnO microcubes	p-n	Two-step solution route	240	Ethanol/50	19.1	5-6	~ 18	[110]
ZnO branched p-Cu_xO@n-ZnO nanowires	p-n	Hydrothermal method and ALD	250	Acetone/5	3.39	62	90	[111]
CuO-In_2O_3 core-shell nanowire	p-n	Thermal oxidation combined with heating	300	CO/960	1.6	25	65	[112]
Fe_2O_3 decorated CuO nanorods	p-n	Solvothermal route	240	Acetone/1000	10.9	64	80	[113]

[a]In this table, the R_a and the R_g were the resistances of the sensor in the air and the resistances of the sensor in the targeted gases, respectively. [b]The NA in the table means that the related information was not given in the references.

Table S4. The assembled strategies of p-p heterojunctions and their gas sensing performances.

Sensing composites	Type of junctions	Synthesis method	Working temperature (°C)	Sensing gas/conc. (ppm)	Sensor response (R_a/R_g) or (R_g/R_a)[a]	Response time (s)	Recovery time (s)	References
TeO_2-core/CuO-shell composite	p-p	Thermal evaporation followed by sputter deposition	150	NO_2/10	4.25	NA	NA	[114]
CuO-NiO core-shell microspheres	p-p	Two-step hydrothermal method	260	H_2S/100	47.6	18	29	[115]
Cu_2O/CuO sub-microspheres	p-p	Hydrothermal treatment	135	H_2S/1	5.5	~ 60	~ 60	[116]
CuO-NiO nanotubes	p-p	one-pot synthesis	110	Glycol/100	10.35	15	45	[117]
$NiFe_2O_4$ nanoparticles-decorated NiO nanosheets	p-p	Solvothermal condition	280	Acetone/50	23	-	-	[118]
NiO/$NiCr_2O_4$ nanoparticles	p-p	Hydrothermal route	225	Xylene/100	66.2	1217	591	[119]
CuO nanoparticles decorated NiO nanosheets	p-p	A simple reflux and hydrothermal method	22	NO_2/100	4.85	2	NA	[120]
$Cu_3Mo_2O_9$@CuO nanorods	p-p	CVD	RT	NO_2/5	1.6	49	241	[121]

[a]In this table, the R_a and the R_g were the resistances of the sensor in the air and the resistances of the sensor in the targeted gases, respectively. [b]The NA in the table means that the related information was not given in the references.

References

[1] Tang: H.; Yan M.; Zhang H.; Li S.; Ma X.; Wang M.; Yang D. A selective NH_3 gas sensor based on Fe_2O_3–ZnO nanocomposites at room temperature. *Sensors and Actuators B: Chemical* **2006**, *114*, 910–915.

[2] Lu, G.; Xu J.; Sun J.; Yu Y.; Zhang Y.; Liu F. UV-enhanced room temperature NO_2 sensor using ZnO nanorods modified with SnO_2 nanoparticles. *Sensors and Actuators B: Chemical* **2012**, *162*, 82–88.

[3] Katoch, A.; Kim J.-H.; Kwon Y.J.; Kim H.W.; Kim S.S. Bifunctional Sensing Mechanism of SnO_2–ZnO Composite Nanofibers for Drastically Enhancing the Sensing Behavior in H_2 Gas. *ACS applied materials & interfaces* **2015**, *7*, 11351–11358.

[4] Hui, G.; Zhu M.; Yang X.; Liu J.; Pan G.; Wang Z. Highly sensitive ethanol gas sensor based on CeO_2/ZnO binary heterojunction composite. *Materials Letters* **2020**, *278*, 128453.

[5] Wang, Z.; Zhi M.; Xu M.; Guo C.; Man Z.; Zhang Z.; Li Q.; Lv Y.; Zhao W.; Yan J.; Zhai C. Ultrasensitive NO_2 gas sensor based on Sb-doped SnO_2 covered ZnO nano-heterojunction. *Journal of Materials Science* **2021**, *56*, 7348–7356.

[6] Wang, L.; Li J.; Wang Y.; Yu K.; Tang X.; Zhang Y.; Wang S.; Wei C. Construction of 1D SnO_2-coated ZnO nanowire heterojunction for their improved n-butylamine sensing performances. *Scientific reports* **2016**, *6*, 35079.

[7] Zou, C.W.; Wang J.; Xie W. Synthesis and enhanced NO_2 gas sensing properties of ZnO nanorods/TiO_2 nanoparticles heterojunction composites. *Journal of Colloid and Interface Science* **2016**, *478*, 22–28.

[8] Lupan, O.; Postica V.; Gröttrup J.; Mishra A.K.; De Leeuw N.H.; Adelung R. Enhanced UV and ethanol vapour sensing of a single 3-D ZnO tetrapod alloyed with Fe_2O_3 nanoparticles. *Sensors and Actuators B: Chemical* **2017**, *245*, 448–461.

[9] Wen, Z.; Tian-Mo L. Gas-sensing properties of SnO_2–TiO_2-based sensor for volatile organic compound gas and its sensing mechanism. *Physica B: Condensed Matter* **2010**, *405*, 1345–1348.

[10] Motaung, D.E.; Mhlongo G.H.; Makgwane P.R.; Dhonge B.P.; Cummings F.R.; Swart H.C.; Ray S.S. Ultra-high sensitive and selective H_2 gas sensor manifested by interface of n–n heterostructure of CeO_2-SnO_2 nanoparticles. *Sensors and Actuators B: Chemical* **2018**, *254*, 984–995.

[11] Gu, D.; Li X.; Zhao Y.; Wang J. Enhanced NO_2 sensing of SnO_2/SnS_2 heterojunction based sensor. *Sensors and Actuators B: Chemical* **2017**, *244*, 67–76.

[12] Qin, S.; Tang P.; Feng Y.; Li D. Novel ultrathin mesoporous ZnO-SnO_2 n-n heterojunction nanosheets with high sensitivity to ethanol. *Sensors and Actuators B: Chemical* **2020**, *309*, 127801.

[13] Xu, H.; Ju J.; Li W.; Zhang J.; Wang J.; Cao B. Superior triethylamine-sensing properties based on TiO_2 /SnO_2 n–n heterojunction nanosheets directly grown on ceramic tubes. *Sensors and Actuators B: Chemical* **2016**, *228*, 634–642.

[14] Xu, H.; Ju D.; Chen Z.; Han R.; Zhai T.; Yu H.; Liu C.; Wu X.; Wang J.; Cao B. A novel hetero-structure sensor based on Au/Mg-doped TiO_2/SnO_2 nanosheets directly grown on Al_2O_3 ceramic tubes. *Sensors and Actuators B: Chemical* **2018**, *273*, 328–335.

[15] Mondal, B.; Basumatari B.; Das J.; Roychaudhury C.; Saha H.; Mukherjee N. ZnO–SnO_2 based composite type gas sensor for selective hydrogen sensing. *Sensors and Actuators B: Chemical* **2014**, *194*, 389–396.

[16] Li, W.; Xu H.; Zhai T.; Yu H.; Chen Z.; Qiu Z.; Song X.; Wang J.; Cao B. Enhanced triethylamine sensing properties by designing Au@SnO_2/MoS_2 nanostructure directly on alumina tubes. *Sensors and Actuators B: Chemical* **2017**, *253*, 97–107.
[17] Choi, K.S.; Park S.; Chang S.-P. Enhanced ethanol sensing properties based on SnO_2 nanowires coated with Fe_2O_3 nanoparticles. *Sensors and Actuators B: Chemical* **2017**, *238*, 871–879.
[18] Van Duy, N.; Van Hieu N.; Huy P.T.; Chien N.D.; Thamilselvan M.; Yi J. Mixed SnO_2/TiO_2 included with carbon nanotubes for gas-sensing application. *Physica E: Low-dimensional Systems and Nanostructures* **2008**, *41*, 258–263.
[19] Chen, Y.-J.; Xiao G.; Wang T.-S.; Zhang F.; Ma Y.; Gao P.; Zhu C.-L.; Zhang E.; Xu Z.; Li Q.-H. α-MoO_3/TiO_2 core/shell nanorods: Controlled-synthesis and low-temperature gas sensing properties. *Sensors and Actuators B: Chemical* **2011**, *155*, 270–277.
[20] Liang, Y.Q.; Cui Z.D.; Zhu S.L.; Li Z.Y.; Yang X.J.; Chen Y.J.; Ma J.M. Design of a highly sensitive ethanol sensor using a nano-coaxial p-Co_3O_4/n-TiO_2 heterojunction synthesized at low temperature. *Nanoscale* **2013**, *5*, 10916–10926.
[21] Deng, J.; Yu B.; Lou Z.; Wang L.; Wang R.; Zhang T. Facile synthesis and enhanced ethanol sensing properties of the brush-like ZnO–TiO_2 heterojunctions nanofibers. *Sensors and Actuators B: Chemical* **2013**, *184*, 21–26.
[22] Wang, Y.; Zhou Y.; Meng C.; Gao Z.; Cao X.; Li X.; Xu L.; Zhu W.; Peng X.; Zhang B.; Lin Y.; Liu L. A high-response ethanol gas sensor based on one-dimensional TiO_2/V_2O_5 branched nanoheterostructures. *Nanotechnology* **2016**, *27*, 425503.
[23] Zhao, P.X.; Tang Y.; Mao J.; Chen Y.X.; Song H.; Wang J.W.; Song Y.; Liang Y.Q.; Zhang X.M. One-Dimensional MoS_2-Decorated TiO_2 nanotube gas sensors for efficient alcohol sensing. *Journal of Alloys and Compounds* **2016**, *674*, 252–258.
[24] Xun, H.; Zhang Z.; Yu A.; Yi J. Remarkably enhanced hydrogen sensing of highly-ordered SnO_2-decorated TiO_2 nanotubes. *Sensors and Actuators B: Chemical* **2018**, *273*, 983–990.
[25] Yu, A.; Xun H.; Yi J. Improving Hydrogen Sensing Performance of TiO_2 Nanotube Arrays by ZnO Modification. *Frontiers in Materials* **2019**, 6,
[26] Chen, K.; Chen S.; Pi M.; Zhang D. SnO_2 nanoparticles/TiO_2 nanofibers heterostructures: In situ fabrication and enhanced gas sensing performance. *Solid State Electron* **2019**, *157*, 42–47.
[27] Nguyen, D.D.; Do D.T.; Vu X.H.; Dang D.V.; Nguyen D.C. ZnO nanoplates surfaced-decorated by WO_3 nanorods for NH_3gas sensing application. *Advances in Natural Sciences: Nanoscience and Nanotechnology* **2016**, *7*, 015004.
[28] Sharma, A.; Tomar M.; Gupta V. WO_3 nanoclusters–SnO_2 film gas sensor heterostructure with enhanced response for NO_2. *Sensors and Actuators B: Chemical* **2013**, *176*, 675–684.
[29] Kida, T.; Nishiyama A.; Hua Z.; Suematsu K.; Yuasa M.; Shimanoe K. WO_3 nanolamella gas sensor: porosity control using SnO_2 nanoparticles for enhanced NO_2 sensing. *Langmuir : the ACS journal of surfaces and colloids* **2014**, *30*, 2571–2579.
[30] Lee, W.I.; Bonyani M.; Lee J.K.; Lee C.; Choi S.-B. Volatile organic compound sensing properties of MoO_3 –ZnO core–shell nanorods. *Current Applied Physics* **2018**, *18*, S60–S67.
[31] Kumar, R.; Goel N.; Mishra M.; Gupta G.; Fanetti M.; Valant M.; Kumar M. Growth of MoS_2–MoO_3 Hybrid Microflowers via Controlled Vapor Transport Process for Efficient Gas Sensing at Room Temperature. *Advanced Materials Interfaces* **2018**, *5*, 1800071.
[32] Li, J.; Liu H.; Fu H.; Xu L.; Jin H.; Zhang X.; Wang L.; Yu K. Synthesis of 1D α-MoO_3/0D ZnO heterostructure nanobelts with enhanced gas sensing properties. *Journal of Alloys and Compounds* **2019**, *788*, 248–256.
[33] Yu, H.-L.; Li L.; Gao X.-M.; Zhang Y.; Meng F.; Wang T.-S.; Xiao G.; Chen Y.-J.; Zhu C.-L. Synthesis and H_2S gas sensing properties of cage-like α-MoO_3/ZnO composite. *Sensors and Actuators B: Chemical* **2012**, 171–172, 679–685.
[34] Jiang, D.; Wei W.; Li F.; Li Y.; Liu C.; Sun D.; Feng C.; Ruan S. Xylene gas sensor based on α-MoO_3/α-Fe_2O_3 heterostructure with high response and low operating temperature. *RSC Advances* **2015**, *5*, 39442–39448.
[35] Mane, A.A.; Nikam S.A.; Moholkar A.V. NO_2 gas sensing properties of sprayed composite porous MoO_3-V_2O_5 thin films. *Mater Chem Phys* **2018**, *216*, 294–304.
[36] Gao, X.; Ouyang Q.; Zhu C.; Zhang X.; Chen Y. Porous MoO_3/SnO_2 Nanoflakes with n–n Junctions for Sensing H_2S. *ACS Applied Nano Materials* **2019**, *2*, 2418–2425.
[37] Xue, D.; Zong F.; Zhang J.; Lin X.; Li Q. Synthesis of Fe_2O_3/WO_3 nanocomposites with enhanced sensing performance to acetone. *Chem Phys Lett* **2019**, *716*, 61–68.
[38] Zhao, C.; Zhang G.; Han W.; Fu J.; He Y.; Zhang Z.; Xie E. Electrospun In_2O_3/α-Fe_2O_3 heterostructure nanotubes for highly sensitive gas sensor applications. *CrystEngComm* **2013**, *15*, 6491.
[39] Kheel, H.; Sun G.-J.; Lee J.K.; Lee S.; Dwivedi R.P.; Lee C. Enhanced H2S sensing performance of TiO_2-decorated α-Fe_2O_3 nanorod sensors. *Ceram Int* **2016**, *42*, 18597–18604.
[40] Si, S.; Li C.; Wang X.; Peng Q.; Li Y. Fe_2O_3/ZnO core–shell nanorods for gas sensors. *Sensors and Actuators B: Chemical* **2006**, *119*, 52–56.
[41] Fan, K.; Guo J.; Cha L.; Chen Q.; Ma J. Atomic layer deposition of ZnO onto Fe_2O_3 nanoplates for enhanced H_2S sensing. *Journal of Alloys and Compounds* **2017**, *698*, 336–340.
[42] Kaneti, Y.V.; Moriceau J.; Liu M.; Yuan Y.; Zakaria Q.; Jiang X.; Yu A. Hydrothermal synthesis of ternary α-Fe_2O_3–ZnO–Au nanocomposites with high gas-sensing performance. *Sensors and Actuators B: Chemical* **2015**, *209*, 889–897.
[43] Wang, L.; Gao J.; Wu B.; Kan K.; Xu S.; Xie Y.; Li L.; Shi K. Designed Synthesis of In_2O_3 Beads@TiO_2-In_2O_3 Composite Nanofibers for High Performance NO_2 Sensor at Room Temperature. *ACS applied materials & interfaces* **2015**, *7*, 27152–27159.
[44] Chi, X.; Liu C.; Liu L.; Li S.; Li H.; Zhang X.; Bo X.; Shan H. Enhanced formaldehyde-sensing properties of mixed Fe_2O_3–In_2O_3 nanotubes. *Mat Sci Semicon Proc* **2014**, *18*, 160–164.
[45] Kim, S.; Park S.; Sun G.-J.; Hyun S.K.; Kim K.-K.; Lee C. Enhanced acetone gas sensing performance of the multiple-networked Fe_2O_3-functionalized In_2O_3 nanowire sensor. *Current Applied Physics* **2015**, *15*, 947–952.
[46] Li, Y.; Deng D.; Xing X.; Chen N.; Liu X.; Xiao X.; Wang Y. A high performance methanol gas sensor based on palladium-platinum-In_2O_3 composited nanocrystalline SnO_2. *Sensors and Actuators B: Chemical* **2016**, *237*, 133–141.

[47] Ma, L.; Fan H.; Tian H.; Fang J.; Qian X. The n-ZnO/n-In_2O_3 heterojunction formed by a surface-modification and their potential barrier-control in methanal gas sensing. *Sensors and Actuators B: Chemical* **2016**, *222*, 508–516.

[48] Park, S.; Kim H.; Jin C.; Lee C. Enhanced gas sensing properties of multiple networked In_2O_3-core/ZnO-shell nanorod sensors. *Journal of nanoscience and nanotechnology* **2013**, *13*, 3427–3432.

[49] Zang, W.; Nie Y.; Zhu D.; Deng P.; Xing L.; Xue X. Core–Shell In_2O_3/ZnO Nanoarray Nanogenerator as a Self-Powered Active Gas Sensor with High H_2S Sensitivity and Selectivity at Room Temperature. *The Journal of Physical Chemistry C* **2014**, *118*, 9209–9216.

[50] Liu, J.; Dai M.; Wang T.; Sun P.; Liang X.; Lu G.; Shimanoe K.; Yamazoe N. Enhanced Gas Sensing Properties of SnO_2 Hollow Spheres Decorated with CeO_2 Nanoparticles Heterostructure Composite Materials. *ACS applied materials & interfaces* **2016**, *8*, 6669–6677.

[51] Lou, Z.; Li F.; Deng J.; Wang L.; Zhang T. Branch-like hierarchical heterostructure (alpha-Fe_2O_3/TiO_2): a novel sensing material for trimethylamine gas sensor. *ACS applied materials & interfaces* **2013**, *5*, 12310–12316.

[52] Da Silva, L.F.; M'peko J.C.; Catto A.C.; Bernardini S.; Mastelaro V.R.; Aguir K.; Ribeiro C.; Longo E. UV-enhanced ozone gas sensing response of ZnO-SnO_2 heterojunctions at room temperature. *Sensors and Actuators B: Chemical* **2017**, *240*, 573–579.

[53] Wei, N.; Cui H.; Wang X.; Xie X.; Wang M.; Zhang L.; Tian J. Hierarchical assembly of In_2O_3 nanoparticles on ZnO hollow nanotubes using carbon fibers as templates: Enhanced photocatalytic and gas-sensing properties. *J Colloid Interface Sci* **2017**, *498*, 263–270.

[54] Liu, Y.; Li G.; Mi R.; Deng C.; Gao P. An environment-benign method for the synthesis of p-NiO/n-ZnO heterostructure with excellent performance for gas sensing and photocatalysis. *Sensors and Actuators B: Chemical* **2014**, *191*, 537–544.

[55] Li, C.; Feng C.; Qu F.; Liu J.; Zhu L.; Lin Y.; Wang Y.; Li F.; Zhou J.; Ruan S. Electrospun nanofibers of p-type NiO/n-type ZnO heterojunction with different NiO content and its influence on trimethylamine sensing properties. *Sensors and Actuators B: Chemical* **2015**, *207*, 90–96.

[56] Hwang, H.; Kim H.; Kim D. Fabrication and Characterization of CuO Nanoparticles/ZnO Nanorods Heterojunction Structure for Room Temperature NO Gas Sensor Application. *Journal of Nanoscience and Nanotechnology* **2016**, *16*, 11608–11612.

[57] Wang, T.; Kou X.; Zhao L.; Sun P.; Liu C.; Wang Y.; Shimanoe K.; Yamazoe N.; Lu G. Flower-like ZnO hollow microspheres loaded with CdO nanoparticles as high performance sensing material for gas sensors. *Sensors and Actuators B: Chemical* **2017**, *250*, 692–702.

[58] Cai, L.; Li H.; Zhang H.; Fan W.; Wang J.; Wang Y.; Wang X.; Tang Y.; Song Y. Enhanced performance of the tangerines-like CuO-based gas sensor using ZnO nanowire arrays. *Materials Science in Semiconductor Processing* **2020**, *118*, 105196.

[59] Wang, X.; Li S.; Xie L.; Li X.; Lin D.; Zhu Z. Low-temperature and highly sensitivity H2S gas sensor based on ZnO/CuO composite derived from bimetal metal-organic frameworks. *Ceramics International* **2020**, *46*, 15858–15866.

[60] Lou, Z.; Deng J.; Wang L.; Wang L.; Fei T.; Zhang T. Toluene and ethanol sensing performances of pristine and PdO-decorated flower-like ZnO structures. *Sensors and Actuators B: Chemical* **2013**, *176*, 323–329.

[61] Park, S.; Sun G.J.; Jin C.; Kim H.W.; Lee S.; Lee C. Synergistic Effects of a Combination of Cr_2O_3-Functionalization and UV-Irradiation Techniques on the Ethanol Gas Sensing Performance of ZnO Nanorod Gas Sensors. *ACS applied materials & interfaces* **2016**, *8*, 2805–2811.

[62] Giebelhaus, I.; Varechkina E.; Fischer T.; Rumyantseva M.; Ivanov V.; Gaskov A.; Morante J.R.; Arbiol J.; Tyrra W.; Mathur S. One-dimensional CuO–SnO_2 p–n heterojunctions for enhanced detection of H_2S. *Journal of Materials Chemistry A* **2013**, *1*, 11261.

[63] Wang, H.; Chen M.; Rong Q.; Zhang Y.; Hu J.; Zhang D.; Zhou S.; Zhao X.; Zhang J.; Zhu Z.; Liu Q. Ultrasensitive xylene gas sensor based on flower-like SnO_2/Co_3O_4 nanorods composites prepared by facile two-step synthesis method. *Nanotechnology* **2020**, *31*, 255501.

[64] Wang, Z.; Li Z.; Sun J.; Zhang H.; Wang W.; Zheng W.; Wang C. Improved Hydrogen Monitoring Properties Based on p-NiO/n-SnO_2 Heterojunction Composite Nanofibers. *The Journal of Physical Chemistry C* **2010**, *114*, 6100–6105.

[65] Ju, D.; Xu H.; Xu Q.; Gong H.; Qiu Z.; Guo J.; Zhang J.; Cao B. High triethylamine-sensing properties of NiO/SnO_2 hollow sphere P–N heterojunction sensors. *Sensors and Actuators B: Chemical* **2015**, *215*, 39–44.

[66] Xue, X.; Xing L.; Chen Y.; Shi S.; Wang Y.; Wang T. Synthesis and H_2S Sensing Properties of CuO−SnO_2 Core/Shell PN-Junction Nanorods. *The Journal of Physical Chemistry C* **2008**, *112*, 12157–12160.

[67] Meng, D.; Liu D.; Wang G.; Shen Y.; San X.; Li M.; Meng F. Low-temperature formaldehyde gas sensors based on NiO-SnO_2 hetero-junction microflowers assembled by thin porous nanosheets. *Sensors and Actuators B: Chemical* **2018**, *273*, 418–428.

[68] Bang, J.H.; Choi M.S.; Mirzaei A.; Kwon Y.J.; Kim S.S.; Kim T.W.; Kim H.W. Selective NO_2 sensor based on Bi_2O_3 branched SnO_2 nanowires. *Sensors and Actuators B: Chemical* **2018**, *274*, 356–369.

[69] Meng, D.; Si J.; Wang M.; Wang G.; Shen Y.; San X.; Meng F. One-step synthesis and the enhanced trimethylamine sensing properties of Co_3O_4/SnO_2 flower-like structures. *Vacuum* **2020**, *171*, 108994.

[70] Chen, Y.-J.; Xiao G.; Wang T.-S.; Zhang F.; Ma Y.; Gao P.; Zhu C.-L.; Zhang E.; Xu Z.; Li Q.-H. Synthesis and enhanced gas sensing properties of crystalline CeO_2/TiO_2 core/shell nanorods. *Sensors and Actuators B: Chemical* **2011**, *156*, 867–874.

[71] Alev, O.; Kilic A.; Cakirlar C.; Buyukkose S.; Ozturk Z.Z. Gas Sensing Properties of p-Co_3O_4/n-TiO_2 Nanotube Heterostructures. *Sensors* **2018**, 18,

[72] Yin, L.; Qu G.; Guo P.; Zhang R.; Sun J.; Chen D. Construction and enhanced low-temperature H_2S-sensing performance of novel hierarchical CuO@WO_3 nanocomposites. *Journal of Alloys and Compounds* **2019**, *785*, 367–373.

[73] Long, H.; Zeng W.; Li T. Hierarchically solvothermal synthesis of WO_3-based nanocomposite: Nature-inspired structure and enhanced gas-sensing property. *Physica E: Low-dimensional Systems and Nanostructures* **2017**, *88*, 206–211.

[74] Ramgir, N.S.; Goyal C.P.; Sharma P.K.; Goutam U.K.; Bhattacharya S.; Datta N.; Kaur M.; Debnath A.K.; Aswal D.K.; Gupta S.K. Selective H_2S sensing characteristics of CuO modified WO_3 thin films. *Sensors and Actuators B: Chemical* **2013**, *188*, 525–532.

[75] Park, S.; Park S.; Jung J.; Hong T.; Lee S.; Kim H.W.; Lee C. H_2S gas sensing properties of CuO-functionalized WO_3 nanowires. *Ceramics International* **2014**, *40*, 11051–11056.

[76] Choi, S.; Bonyani M.; Sun G.-J.; Lee J.K.; Hyun S.K.; Lee C. Cr_2O_3 nanoparticle-functionalized WO_3 nanorods for ethanol gas sensors. *Applied Surface Science* **2018**, *432*, 241–249.

[77] He, M.; Xie L.; Zhao X.; Hu X.; Li S.; Zhu Z.-G. Highly sensitive and selective H_2S gas sensors based on flower-like WO_3/CuO composites operating at low/room temperature. *Journal of Alloys and Compounds* **2019**, *788*, 36–43.
[78] Wang, T.-S.; Wang Q.-S.; Zhu C.-L.; Ouyang Q.-Y.; Qi L.-H.; Li C.-Y.; Xiao G.; Gao P.; Chen Y.-J. Synthesis and enhanced H2S gas sensing properties of α-MoO_3/CuO p–n junction nanocomposite. *Sensors and Actuators B: Chemical* **2012**, 171–172, 256–262.
[79] Annanouch, F.E.; Roso S.; Haddi Z.; Vallejos S.; Umek P.; Bittencourt C.; Blackman C.; Vilic T.; Llobet E. p-Type PdO nanoparticles supported on n-type WO_3 nanoneedles for hydrogen sensing. *Thin Solid Films* **2016**, *618*, 238–245.
[80] Mirzaei, A.; Park S.; Sun G.-J.; Kheel H.; Lee C.; Lee S. Fe_2O_3/Co_3O_4 composite nanoparticle ethanol sensor. *J Korean Phys Soc* **2016**, *69*, 373–380.
[81] Huang, X.-Y.; Chi Z.-T.; Liu J.; Li D.-H.; Sun X.-J.; Yan C.; Wang Y.-C.; Li H.; Wang X.-D.; Xie W.-F. Enhanced gas sensing performance based on p-NiS/n-In_2O_3 heterojunction nanocomposites. *Sensors and Actuators B: Chemical* **2020**, *304*, 127305.
[82] Park, S.; Kim S.; Sun G.-J.; Lee C. Synthesis, Structure, and Ethanol Gas Sensing Properties of In_2O_3 Nanorods Decorated with Bi_2O_3 Nanoparticles. *ACS applied materials & interfaces* **2015**, *7*, 8138–8146.
[83] Yan, C.; Lu H.; Gao J.; Zhu G.; Yin F.; Yang Z.; Liu Q.; Li G. Synthesis of porous NiO-In_2O_3 composite nanofibers by electrospinning and their highly enhanced gas sensing properties. *Journal of Alloys and Compounds* **2017**, *699*, 567–574.
[84] Kim, J.-H.; Lee J.-H.; Mirzaei A.; Kim H.W.; Kim S.S. Optimization and gas sensing mechanism of n-SnO_2-p-Co_3O_4 composite nanofibers. *Sensors and Actuators B: Chemical* **2017**, *248*, 500–511.
[85] Park, S.; Kim S.; Kheel H.; Lee C. Oxidizing gas sensing properties of the n-ZnO/p-Co_3O_4 composite nanoparticle network sensor. *Sensors and Actuators B: Chemical* **2016**, *222*, 1193–1200.
[86] Huang, J.; Dai Y.; Gu C.; Sun Y.; Liu J. Preparation of porous flower-like CuO/ZnO nanostructures and analysis of their gas-sensing property. *Journal of Alloys and Compounds* **2013**, *575*, 115–122.
[87] Kim, J.; Kim W.; Yong K. CuO/ZnO Heterostructured Nanorods: Photochemical Synthesis and the Mechanism of H_2S Gas Sensing. *The Journal of Physical Chemistry C* **2012**, *116*, 15682–15691.
[88] Patil, L.A.; Patil D.R. Heterocontact type CuO-modified SnO_2 sensor for the detection of a ppm level H_2S gas at room temperature. *Sensors and Actuators B: Chemical* **2006**, *120*, 316–323.
[89] Shao, F.; Hoffmann M.W.G.; Prades J.D.; Zamani R.; Arbiol J.; Morante J.R.; Varechkina E.; Rumyantseva M.; Gaskov A.; Giebelhaus I.; Fischer T.; Mathur S.; Hernández-Ramírez F. Heterostructured p-CuO (nanoparticle)/n-SnO_2 (nanowire) devices for selective H_2S detection. *Sensors and Actuators B: Chemical* **2013**, *181*, 130–135.
[90] Wang, L.; Kang Y.; Wang Y.; Zhu B.; Zhang S.; Huang W.; Wang S. CuO nanoparticle decorated ZnO nanorod sensor for low-temperature H_2S detection. *Materials Science and Engineering: C* **2012**, *32*, 2079–2085.
[91] Yu, M.-R.; Suyambrakasam G.; Wu R.-J.; Chavali M. Performance evaluation of ZnO–CuO hetero junction solid state room temperature ethanol sensor. *Mater Res Bull* **2012**, *47*, 1713–1718.
[92] Zhang, Y.-B.; Yin J.; Li L.; Zhang L.-X.; Bie L.-J. Enhanced ethanol gas-sensing properties of flower-like p-CuO/n-ZnO heterojunction nanorods. *Sensors and Actuators B: Chemical* **2014**, *202*, 500–507.
[93] Liu, T.; Yu Z.; Liu Y.; Gao J.; Wang X.; Suo H.; Yang X.; Zhao C.; Liu F. Gas sensor based on Ni foam: SnO_2-decorated NiO for Toluene detection. *Sensors and Actuators B: Chemical* **2020**, *318*, 128167.
[94] Dey, S.; Nag S.; Santra S.; Ray S.K.; Guha P.K. Voltage-controlled NiO/ZnO p–n heterojunction diode: a new approach towards selective VOC sensing. *Microsystems & Nanoengineering* **2020**, *6*, 35.
[95] Wang, Z.; Zhang K.; Fei T.; Gu F.; Han D. α-Fe_2O_3/NiO heterojunction nanorods with enhanced gas sensing performance for acetone. *Sensors and Actuators B: Chemical* **2020**, *318*, 128191.
[96] Wang, C.; Cheng X.; Zhou X.; Sun P.; Hu X.; Shimanoe K.; Lu G.; Yamazoe N. Hierarchical alpha-Fe_2O_3/NiO composites with a hollow structure for a gas sensor. *ACS applied materials & interfaces* **2014**, *6*, 12031–12037.
[97] Park, S.; Kim S.; Sun G.-J.; Choi S.; Lee S.; Lee C. Ethanol sensing properties of networked In_2O_3 nanorods decorated with Cr_2O_3-nanoparticles. *Ceram Int* **2015**, *41*, 9823–9827.
[98] Park, S.; Sun G.-J.; Kheel H.; Lee W.I.; Lee S.; Choi S.-B.; Lee C. Synergistic effects of codecoration of oxide nanoparticles on the gas sensing performance of In_2O_3 nanorods. *Sensors and Actuators B: Chemical* **2016**, *227*, 591–599.
[99] Bekermann, D.; Gasparotto A.; Barreca D.; Maccato C.; Comini E.; Sada C.; Sberveglieri G.; Devi A.; Fischer R.A. Co_3O_4/ZnO nanocomposites: from plasma synthesis to gas sensing applications. *ACS applied materials & interfaces* **2012**, *4*, 928–934.
[100] Cao, J.; Wang Z.; Wang R.; Liu S.; Fei T.; Wang L.; Zhang T. Core–shell Co_3O_4/α-Fe_2O_3 heterostructure nanofibers with enhanced gas sensing properties. *RSC Advances* **2015**, *5*, 36340–36346.
[101] Jeong, H.M.; Kim J.H.; Jeong S.Y.; Kwak C.H.; Lee J.H. Co_3O_4-SnO_2 Hollow Heteronanostructures: Facile Control of Gas Selectivity by Compositional Tuning of Sensing Materials via Galvanic Replacement. *ACS applied materials & interfaces* **2016**, *8*, 7877–7883.
[102] Qu, F.; Liu J.; Wang Y.; Wen S.; Chen Y.; Li X.; Ruan S. Hierarchical Fe_3O_4@Co_3O_4 core–shell microspheres: Preparation and acetone sensing properties. *Sensors and Actuators B: Chemical* **2014**, *199*, 346–353.
[103] Staerz, A.; Kim T.-H.; Lee J.-H.; Weimar U.; Barsan N. Nanolevel Control of Gas Sensing Characteristics via p–n Heterojunction between Rh_2O_3 Clusters and WO_3 Crystallites. *The Journal of Physical Chemistry C* **2017**, *121*, 24701–24706.
[104] Wang, L.; Lou Z.; Zhang R.; Zhou T.; Deng J.; Zhang T. Hybrid Co_3O_4/SnO_2 Core-Shell Nanospheres as Real-Time Rapid-Response Sensors for Ammonia Gas. *ACS applied materials & interfaces* **2016**, *8*, 6539–6545.
[105] Xiong, Y.; Xu W.; Zhu Z.; Xue Q.; Lu W.; Ding D.; Zhu L. ZIF-derived porous ZnO-Co_3O_4 hollow polyhedrons heterostructure with highly enhanced ethanol detection performance. *Sensors and Actuators B: Chemical* **2017**, *253*, 523–532.
[106] Zhang, L.; Jing X.; Liu J.; Wang J.; Sun Y. Facile synthesis of mesoporous ZnO/Co_3O_4 microspheres with enhanced gas-sensing for ethanol. *Sensors and Actuators B: Chemical* **2015**, *221*, 1492–1498.
[107] B., Z.; M. L.; Z. S.; H. K.; H. Y.; Q. L.; G. Z.; H. L. *Sens. Actuators, B* **2017**, *249*, 558.

[108] Chowdhuri, A.; Gupta V.; Sreenivas K. Fast response H_2S gas sensing characteristics with ultra-thin CuO islands on sputtered SnO_2. *Sensors and Actuators B: Chemical* **2003**, *93*, 572–579.
[109] Mridha, S.; Basak D. Investigation of a p-CuO/n-ZnO thin film heterojunction for H2gas-sensor applications. *Semiconductor Science and Technology* **2006**, *21*, 928–932.
[110] Yin, M.; Wang F.; Fan H.; Xu L.; Liu S. Heterojunction CuO@ZnO microcubes for superior p-type gas sensor application. *Journal of Alloys and Compounds* **2016**, *672*, 374–379.
[111] Xue, X.-T.; Zhu L.-Y.; Yuan K.-P.; Zeng C.; Li X.-X.; Ma H.-P.; Lu H.-L.; Zhang D.W. ZnO branched p-CuxO @n-ZnO heterojunction nanowires for improving acetone gas sensing performance. *Sensors and Actuators B: Chemical* **2020**, *324*, 128729.
[112] Li, X.; Li X.; Chen N.; Li X.; Zhang J.; Yu J.; Wang J.; Tang Z. CuO-In_2O_3Core-Shell Nanowire Based Chemical Gas Sensors. *Journal of Nanomaterials* **2014**, 2014, 1–7.
[113] Park, S.; Kheel H.; Sun G.-J.; Ko T.; Lee W.I.; Lee C. Acetone Gas Sensing Properties of a Multiple-Networked Fe_2O_3-Functionalized CuO Nanorod Sensor. *Journal of Nanomaterials* **2015**, 2015, 1–6.
[114] Park, S.; Kim S.; Sun G.-J.; In Lee W.; Kim K.K.; Lee C. Fabrication and NO_2 gas sensing performance of TeO_2-core/CuO-shell heterostructure nanorod sensors. *Nanoscale Res Lett* **2014**, *9*, 638.
[115] Wang, Y.; Qu F.; Liu J.; Wang Y.; Zhou J.; Ruan S. Enhanced H_2S sensing characteristics of CuO-NiO core-shell microspheres sensors. *Sensors and Actuators B: Chemical* **2015**, *209*, 515–523.
[116] Meng, F.-N.; Di X.-P.; Dong H.-W.; Zhang Y.; Zhu C.-L.; Li C.; Chen Y.-J. Ppb H_2S gas sensing characteristics of Cu_2O/CuO sub-microspheres at low-temperature. *Sensors and Actuators B: Chemical* **2013**, *182*, 197–204.
[117] Su, C.; Zhang L.; Han Y.; Ren C.; Zeng M.; Zhou Z.; Su Y.; Hu N.; Wei H.; Yang Z. Controllable synthesis of heterostructured CuO–NiO nanotubes and their synergistic effect for glycol gas sensing. *Sensors and Actuators B: Chemical* **2020**, *304*, 127347.
[118] Xu, Y.; Fan Y.; Tian X.; Liang Q.; Liu X.; Sun Y. p-p heterojunction composite of $NiFe_2O_4$ nanoparticles-decorated NiO nanosheets for acetone gas detection. *Materials Letters* **2020**, *270*, 127728.
[119] Gao, H.; Guo J.; Li Y.; Xie C.; Li X.; Liu L.; Chen Y.; Sun P.; Liu F.; Yan X.; Liu F.; Lu G. Highly selective and sensitive xylene gas sensor fabricated from NiO/$NiCr_2O_4$ p-p nanoparticles. *Sensors and Actuators B: Chemical* **2019**, *284*, 305–315.
[120] Xu, H.; Zhang J.; Rehman A.U.; Gong L.; Kan K.; Li L.; Shi K. Synthesis of NiO@CuO nanocomposite as high-performance gas sensing material for NO_2 at room temperature. *Applied Surface Science* **2017**, *412*, 230–237.
[121] Adamu, B.I.; Falak A.; Tian Y.; Tan X.; Meng X.; Chen P.; Wang H.; Chu W. p–p Heterojunction Sensors of p-$Cu_3Mo_2O_9$ Micro/Nanorods Vertically Grown on p-CuO Layers for Room-Temperature Ultrasensitive and Fast Recoverable Detection of NO_2. *ACS Applied Materials & Interfaces* **2020**, *12*, 8411–8421.
